# STIM and Orai Mediated Regulation of Calcium Signaling in Age-Related Diseases

**DOI:** 10.3389/fragi.2022.876785

**Published:** 2022-04-19

**Authors:** Helen E. Collins, Dingguo Zhang, John C. Chatham

**Affiliations:** ^1^ Division of Environmental Medicine, Department of Medicine, University of Louisville, Louisville, KY, United States; ^2^ Division of Molecular and Cellular Pathology, Department of PathologyUniversity of Alabama at Birmingham, Birmingham, AL, United States

**Keywords:** calcium, neurodegeneration, cardiovascular disease, STIM, orai, cell survival, SOCE channels

## Abstract

Tight spatiotemporal regulation of intracellular Ca^2+^ plays a critical role in regulating diverse cellular functions including cell survival, metabolism, and transcription. As a result, eukaryotic cells have developed a wide variety of mechanisms for controlling Ca^2+^ influx and efflux across the plasma membrane as well as Ca^2+^ release and uptake from intracellular stores. The STIM and Orai protein families comprising of STIM1, STIM2, Orai1, Orai2, and Orai3, are evolutionarily highly conserved proteins that are core components of all mammalian Ca^2+^ signaling systems. STIM1 and Orai1 are considered key players in the regulation of Store Operated Calcium Entry (SOCE), where release of Ca^2+^ from intracellular stores such as the Endoplasmic/Sarcoplasmic reticulum (ER/SR) triggers Ca^2+^ influx across the plasma membrane. SOCE, which has been widely characterized in non-excitable cells, plays a central role in Ca^2+^-dependent transcriptional regulation. In addition to their role in Ca^2+^ signaling, STIM1 and Orai1 have been shown to contribute to the regulation of metabolism and mitochondrial function. STIM and Orai proteins are also subject to redox modifications, which influence their activities. Considering their ubiquitous expression, there has been increasing interest in the roles of STIM and Orai proteins in excitable cells such as neurons and myocytes. While controversy remains as to the importance of SOCE in excitable cells, STIM1 and Orai1 are essential for cellular homeostasis and their disruption is linked to various diseases associated with aging such as cardiovascular disease and neurodegeneration. The recent identification of splice variants for most STIM and Orai isoforms while complicating our understanding of their function, may also provide insight into some of the current contradictions on their roles. Therefore, the goal of this review is to describe our current understanding of the molecular regulation of STIM and Orai proteins and their roles in normal physiology and diseases of aging, with a particular focus on heart disease and neurodegeneration.

## 1 Introduction

It is universally recognized that tight spatiotemporal regulation of cytoplasmic Ca^2+^ is essential for cellular homeostasis and that dysregulation of Ca^2+^ signaling is associated with the development of pathophysiology. Homologs of human plasma membrane Ca^2+^ channels have been found in organisms as distant as the protozoan *Naegleria gruberi* demonstrating that regulation of extracellular influx as a Ca^2+^ signaling mechanism has existed for over 1 billion years (Collins and Meyer, 2011). Eukaryotic cells have developed evolutionary highly conserved mechanisms for controlling Ca^2+^ influx and efflux across the plasma membrane, and Ca^2+^ release and uptake from intracellular stores, such as the endoplasmic reticulum (ER).

In the late 1970s, Putney reported a potential link between the transient release of Ca^2+^ from intracellular stores to subsequent influx of extracellular Ca^2+^ (Putney, 1977). The biophysics underlying this phenomenon, which subsequently became known as store-operated Ca^2+^ entry (SOCE), became increasingly well characterized over the following two decades (Parekh and Putney, 2005). It was found that physiologically, Ca^2+^ release from ER/SR was triggered in an agonist-dependent manner, typically, although not exclusively, via inositol 1,4,5-trisphosphate (IP_3_)-mediated activation of the IP_3_ receptor (IP_3_R). This was followed by the activation of a highly selective non-voltage gated, Ca^2+^ channel in the plasma membrane. In contrast to IP_3_-induced release of Ca^2+^ from intracellular stores, which results in transient increases in Ca^2+^ of the order of seconds or less, SOCE can remain active for minutes or longer (Soboloff et al., 2012). The longer duration of SOCE is an important factor in its role in Ca^2+^-dependent regulation of gene transcription, such as the canonical Ca^2+^/calmodulin-dependent activation of the phosphatase calcineurin, followed by dephosphorylation and nuclear translocation of transcription factors such as nuclear factor of activated T cells (NFAT) and nuclear factor kappa B (NF-κB) (Parekh and Putney, 2005). However, the identity of the molecular mediators of SOCE remained elusive until a remarkable series of papers published in 2005 and 2006 identified Stromal Interaction Molecule-1 (STIM1) and the Calcium Release-Activated Calcium Modulator 1 (CRACM1, now known as Orai1) as the ER/SR Ca^2+^ sensor and the plasma membrane Ca^2+^ channel respectively, that together regulated SOCE (Roos et al., 2005; Zhang et al., 2005; Vig et al., 2006b; Feske et al., 2006; Mercer et al., 2006; Peinelt et al., 2006; Prakriya et al., 2006; Soboloff et al., 2006; Taylor, 2006; Yeromin et al., 2006; Zhang et al., 2006).

Since their identification, STIM1 and Orai1 have been widely accepted as being essential components of SOCE. As discussed below, the detailed molecular interactions between the two proteins required to facilitate SOCE have been elucidated; however, the role of their homologs STIM2, Orai2, and Orai3 remain poorly understood. To complicate matters further, several variants of STIM1, STIM2, Orai1, and Orai2 have also been identified (Gross et al., 2007; Darbellay et al., 2011; Fukushima et al., 2012; Miederer et al., 2015; Rana et al., 2015; Knapp et al., 2020; Ramesh et al., 2021). While the molecular mechanisms underlying the regulation of SOCE have been almost exclusively studied in non-excitable cells, the expression of STIM1 and Orai1 is ubiquitous, and consequently they are also found in excitable cells including myocytes and neurons. However, ongoing controversies regarding the presence of SOCE in excitable cells has suggested possible non-canonical functions of STIM1, Orai1, and their homologs in such cells. Therefore, the goal of this review is to provide a thorough understanding of the molecular regulation of STIM and Orai proteins, their roles in normal physiology. We also discuss their roles in regulating mitochondrial function and metabolism, redox regulation, and cell survival mechanisms—all of which are components of normal healthy aging. Much of the work on the roles of STIM and Orai has been focused on non-excitable cells, particularly that related to the immune system; however, there is growing evidence that they are also involved in regulating the function of excitable cells such as neurons and cardiomyocytes. Therefore, we have also discussed the contributions of defects in STIM and Orai function in key age-related diseases such as cardiovascular disease and neurodegeneration. We have also summarized the few studies that have examined the potential roles of STIM and Orai dysfunction in the normal aging process.

## 2 STIMs—Gene and Protein Structures

### 2.1 STIM1

In 2005 two independent studies, both using siRNA arrays, identified for the first time, that STIM1 played a central role in mediating SOCE (Liou et al., 2005; Roos et al., 2005). In 1996 there were two reports describing a protein of unknown function, one identified a gene called *GOK* that was predicted to encode a protein that contained a transmembrane helix (Parker et al., 1996), the other identified a stromal interacting molecule (SIM) (Oritani and Kincade, 1996). SIM and GOK were subsequently named STIM1. Some lines of evidence suggested it might be a tumor suppressor gene (Parker et al., 1996; Sabbioni et al., 1997), but its function remained elusive. Early studies correctly characterized STIM1 as a type 1 transmembrane protein that was widely expressed and highly conserved. It was also shown to be phosphorylated in the C-terminal region, a possible target for mitogen-activated protein kinases (MAPK), and initially identified as cell surface protein (Manji et al., 2000). In addition, it was recognized that the N-terminal region contained consensus sequences for EF-hand calcium binding motifs (Williams et al., 2001). In 2005, in addition to demonstrating that STIM1 was essential for SOCE, Liou *et al.* reported that STIM1 was located primarily in the ER (Liou et al., 2005). Moreover, they also showed that ER Ca^2+^ depletion resulted in the redistribution of STIM1 into puncta that were close to the plasma membrane, and that this redistribution of STIM1 occurred because its EF-hand motifs sensed decreases in ER Ca^2+^ (Liou et al., 2005). These fundamental observations regarding STIM1 function, were confirmed later the in same year by Zhang *et al.* (Zhang et al., 2005). Although predominantly located in the ER, depending on cell type and cell cycle, 5–20% of STIM1 is also found at the plasma membrane (Mignen et al., 2007; Hewavitharana et al., 2008; Ercan et al., 2012).

#### 2.1.1 STIM1 structure

**FIGURE 1:**
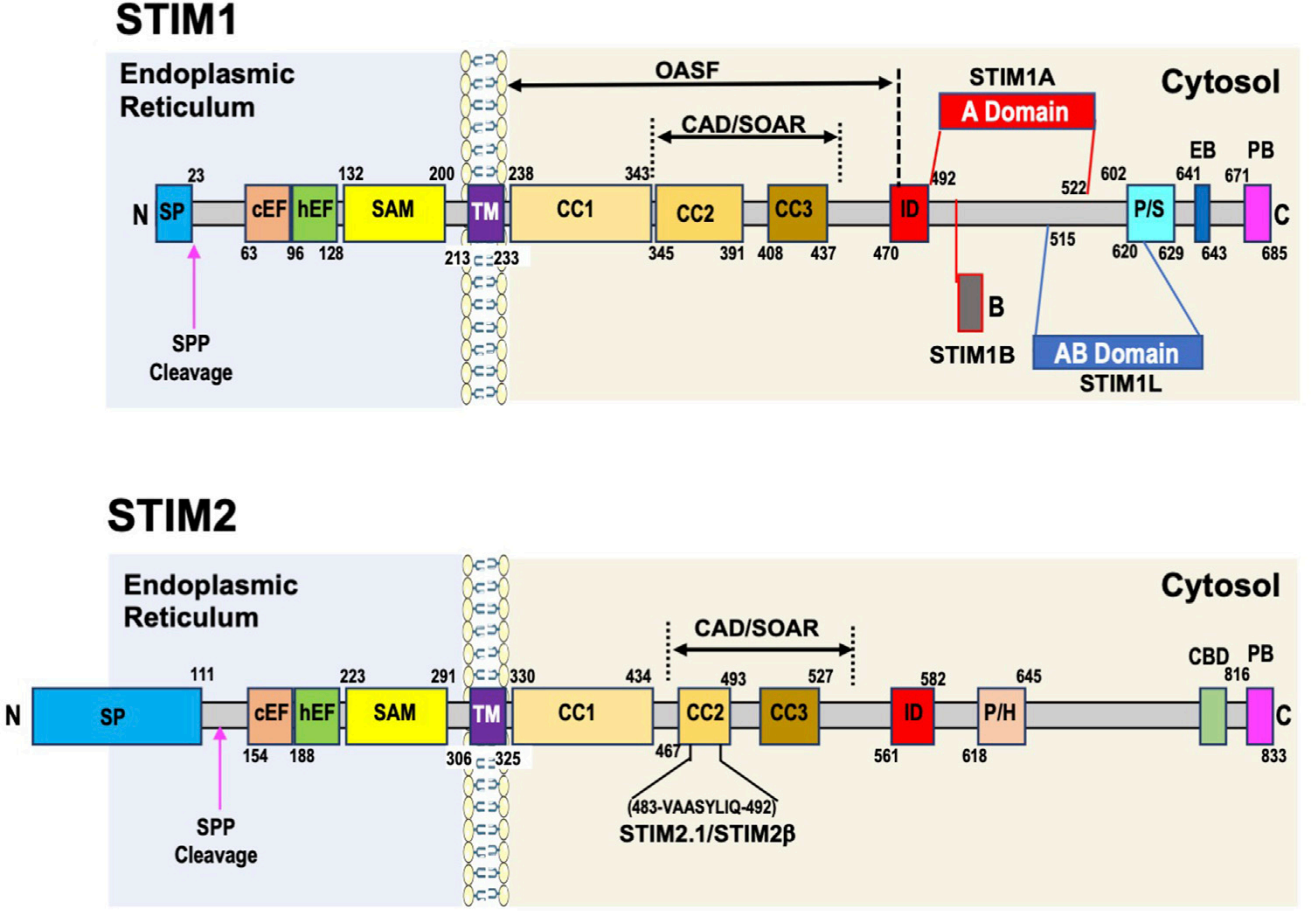
Domain structure of human STIM1 and STIM2 proteins. STIM1 has a short signaling peptide (SP) at the N-terminus which is cleaved by a signaling peptide peptidase (SPP) when the protein is localized to ER membrane. In the endoplasmic reticulum the key domains are canonical and hidden EF hand domains (cEF, hEF) and the sterile alpha motif (SAM domain. These regions play a critical role in regulating SOCE, with the cEF domain as the sensor of ER Ca^2+^, and the hEF and SAM domains contributing to the initial conformation changes that are transmitted via the transmembrane domain (TM) to coiled coil (CC)1 domain, which releases the CAD/SOAR region (CRAC-activating domain/STIM-Orai-activating region), which includes CC2 and CC3 domain, facilitating both the extension of the C-terminal domain towards the plasma membrane and STIM1 oligomerization, both of which are required for SOCE activation. Other important cytosolic domains include the inhibitory domain (ID), which is involved in Ca^2+^ dependent inhibition of SOCE; the proline/serine domain (P/S), which contains many phosphorylation sites that can regulate STIM1 function; the End binding protein domain (EB) and the polybasic domain (PB) at the C-terminus. STIM1L is a splice variant found predominantly in striated muscle includes the insertion of an actin binding domain (ABD) between aa515 and aa620. STIM1A is another splice variant with insertion of an A domain just after the ID domain between aa492 and 522. STIM2 architecture overall is very similar to STIM1 with a few key differences. Like STIM1 it has cEF, hEF, and SAM domains in the endoplasmic reticulum, a TM domain, followed by CC1, CC2, and CC3 domains in the cytosol with a PB region at the C-terminus. As described in the text subtle differences in these regions lead to changes in function compared to STIM1. Differences with STIM1 include an unusually long signaling peptide region of ∼100aa, a proline/histidine region (P/H), and a calmodulin binding domain (CBD) close to the PB region. It is unclear whether STIM2 contains an ID region. There are two known STIM2 splice variants, one STIM2.1 also known as STIM2β involves the insertion of an 8 amino acid sequence (VAASYLIQ) within CC2 domain.

The domain structure of mammalian STIM1 (Figure 1) is characterized by an ER signal peptide, followed by a canonical EF-hand (cEF) Ca^2+^ binding domain in the N-terminal region of the protein. The cEF-hand domain localized to the lumen of the ER (Gudlur et al., 2020), is critical to the Ca^2+^ sensing function of STIM1. Mutations in the cEF region of STIM1 decrease its sensitivity to ER Ca^2+^ concentrations and result in a constitutively active STIM1 (Liou et al., 2005; Zhang et al., 2005; Spassova et al., 2006). The cEF domain is followed by a hidden or non-canonical EF hand (hEF or ncEF), which does not bind Ca^2+^ and a sterile alpha-motif (SAM) domain (Stathopulos et al., 2008). The combined EF-SAM domains are key to regulating SOCE, with the cEF domain as the sensor of EF Ca^2+^ and the hEF domain essential for regulating the stability of the EF-SAM region. The transmembrane domain (TM) connects the ER and cytosolic regions of STIM1. In the Ca^2+^ bound state, the EF-SAM domains on STIM1 are kept apart to prevent spontaneous activation.

While the ER luminal region of the protein is critical for initiating the response of STIM1 to changes in ER Ca^2+^ levels, Huang *et al.*, demonstrated that the cytoplasmic carboxy-terminal domain of STIM1 was sufficient to activate Ca^2+^ entry in the absence of store depletion (Huang et al., 2006). Immediately following the TM domain are three coiled coil domains CC1, CC2, and CC3. An Orai1 activating STIM1 fragment (OASF) (Muik et al., 2009) as well as a minimal region required for gaiting Orai1 channels, the STIM1-Orai activating region (SOAR) also known as the Ca^2+^-release-activated Ca^2+^ (CRAC) activation domain (CAD) were identified in the CC domains (Derler et al., 2016a; Lewis, 2020). The OASF spans all three CC domains while the CAD/SOAR region encompasses CC2 and CC3 domains. The CAD/SOAR region is divided into four helices, the first, Sα1, corresponds to CC2 and the last Sα4 to CC3, with Sα2 and Sα3 located between CC2 and CC3 domains (Yang et al., 2012). A mutation of a single amino acid Phe394 to histidine, in the Sα2 helix of the SOAR domain, completely prevented STIM1 activation of Orai1 (Wang et al., 2014). The Sα3 helical segment while not involved with STIM1 colocalization with Orai1 is essential for activating channel opening (Butorac et al., 2019). The CC2 component of the SOAR domain has been shown to contain a cholesterol binding region, which following store depletion binds cholesterol, acting as a negative regulator of SOCE (Pacheco et al., 2016).

When the SR Ca^2+^ stores are full (i.e., 1 to 5 × 10^−4^ M (Bagur and Hajnoczky, 2017)) the CC1 domain interacts with the SOAR/CAD region keeping it in an inactivated state by clamping it close to the ER membrane (Lewis, 2020). The CC1 domains contain 3 CC regions CC1α_1_, CC1α_2_, and CC1α_3_ and evidence suggests that the CC1α_1_ and CC1α_3_ regions, in combination with the CC2 and CC3 domains, play a key role in keeping the SOAR/CAD domain in the inactivated state (Fahrner et al., 2014). In response to store depletion, reorganization of the EF-SAM region coupled with reorientation of both the transmembrane helices leads to the homomerization of CC1α_1_ region and release of SOAR/CAD domain from its inactive state (Fahrner et al., 2020). The importance of the TM domain in contributing to this initial STIM1 conformational change was demonstrated by gain of function mutations in this region leading to constitutive STIM1 puncta formation and Ca^2+^ influx (Ma et al., 2015). The subsequent reorientation of the CC1α_1_, CC1α_2_, and CC1α_3_ regions in the CC1 domain not only helps to extend the SOAR/CAD domain towards the plasma membrane but also contributes to the oligomerization of STIM1 necessary for activation of SOCE (Fahrner et al., 2020). Of note however it is only the CC1α_1_ region that is essential for activating SOCE (Fahrner et al., 2014). The CC3 domains also contributes to STIM1 oligomerization leading to larger STIM1 clusters (Fahrner et al., 2014); the region aa420-450 of the CC3 domain has been described as a STIM1 homomerization domain (SHD) (Muik et al., 2009). The resulting extension of the STIM1 cytoplasmic section, enables the short polybasic (PB) region at the C-terminus to interact with plasma membrane phospholipids thereby partly facilitating the localization of STIM1 to ER-PM junctions. This is supported by the observation that deletion of this region decreases the size of STIM1 plasma membrane clusters that form following ER Ca^2+^ depletion (Maleth et al., 2014; Sauc et al., 2015). While the PB region is not essential for SOCE it appears to improve the efficiency with which STIM1 interacts with Orai1 (Lewis, 2020). Another important C-terminal regulatory domain is the Inhibitory domain (ID). Like other Ca^2+^-channels, the STIM1-Orai1 channel is inhibited by Ca^2+^ in a feedback manner that occurs in a time frame of milliseconds. This process, called Ca^2+^ dependent inhibition (CDI), requires the ID domain although this domain itself is not the primary Ca^2+^ sensor for CDI of SOCE (Mullins and Lewis, 2016). Full CDI requires the interaction of the ID with key Orai1 tryptophan and tyrosine residues. Early studies suggested that calmodulin, similar to its role in regulating CDI in voltage gated Ca^2+^ channels, was the SOCE Ca^2+^ sensor for CDI (Mullins et al., 2009; Liu Y. et al., 2012); however, subsequent studies indicated that this was not the case (Mullins et al., 2016). Calmodulin has been implicated in a slower Ca^2+^-dependent inactivation process via interaction with the SOAR/CAD domain facilitating dissociation between STIM1 and Orai1 (Li et al., 2017).

#### 2.1.2 STIM1 regulation

Additional key regulatory domains in STIM1 are the end binding protein1 (EB1) domain and the Proline/Serine rich region (P/S). In 2008 Grigoriev *et al.*, identified STIM1 as an microtubule associated protein RP/EB family member 1 (EB1) interacting protein; however, the function of this interaction was unclear as loss of EB1 had no effect on SOCE (Grigoriev et al., 2008). More recent studies have shown that EB1 dynamically traps STIM1 thereby limiting excess STIM1 in ER-PM junctions, potentially preventing ER Ca^2+^ overload (Chang et al., 2018). EB1 is a microtubule plus-end tracking protein (+TIP) and is recognized as a master regulator of +TIP function and thus microtubule dynamics (Akhmanova and Steinmetz, 2008). STIM1 has also been identified as a +TIP and its EB domain contains a Thr-Arg-Ile-Pro sequence (TRIP), a motif common to other EB1 binding proteins (Akhmanova and Steinmetz, 2008; Grigoriev et al., 2008). Phosphorylation in regions adjacent to the EB/TRIP domain negatively regulate the interactions of +TIP with EB1 (Smyth et al., 2012). Of note, the P/S region of STIM1 is close to the STIM1 EB/TRIP domain, and phosphorylation of Ser575, Ser608, and Ser621 in that region by extracellular signal-regulated kinases 1/2 (ERK1/2) regulates the interactions between STIM1 and EB1, which is required for activation of SOCE (Pozo-Guisado and Martin-Romero, 2013). On the other hand, phosphorylation of Ser668 by cyclin dependent kinase 1 (CDK1) has been implicated in inactivation of SOCE during mitosis (Smyth et al., 2009). To date over 30 STIM1 phosphorylation sites have been mapped many of which are located in or adjacent to the P/S region (Hornbeck et al., 2004). Phosphorylation outside of the P/S domain has also been shown to modulate STIM1 function. For example, in endothelial cells ER Ca^2+^ depletion leads to phosphorylation of Tyr361 in the SOAR/CAD domain by proline rich kinase 2 (Pyk2) thereby facilitating SOCE (Yazbeck et al., 2017). AMPK phosphorylates STIM1 at Ser257, located in the CC1 domain, and phosphorylation of this site favors an inactive STIM1 conformation (Nelson et al., 2019). PKA phosphorylates Thr389 regulating a non-SOCE function of STIM1 (Thompson and Shuttleworth, 2015) and dual-specificity tyrosine phosphorylation-regulated kinase (DYRK2) phosphorylates Ser519 and Ser521, enhancing STIM1 and Orai1 interactions (Wei et al., 2021). A number of phosphorylation sites have also been identified in the N-terminal luminal domain of STIM1 (Hornbeck et al., 2004); however their function and kinases are not known.

STIM1 is also subject to oxidative modifications that affect its function. For example, Cys56 in the STIM1 luminal region is subjected to S-glutathionylation in response to oxidant stress, resulting in constitutive Ca^2+^ entry independent of Ca^2+^ store levels (Hawkins et al., 2010). Both Cys49 and Cys56 undergo nitric oxide (NO)-mediated S-nitrosylation, which resulted in stabilization of the EF-SAM region inhibiting SOCE (Gui et al., 2018). The modification of serine and threonine residues with O-linked N-acetylglucosamine (O-GlcNAc) is increasing recognized as an important nutrient mediated signaling mechanism (Chatham et al., 2021). STIM1 has been shown to be O-GlcNAcylated and pharmacologically mediated increases in O-GlcNAc attenuated STIM1 puncta formation and SOCE (Zhu-Mauldin et al., 2012). Nomura *et al.*, reported that Ser621 and Thr626 in STIM1 were O-GlcNAcylated (Nomura et al., 2020). They observed that decreased O-GlcNAcylation at Thr626 and increased O-GlcNAcylation at Ser621 both attenuated SOCE, possibly by decreasing Ser621 phosphorylation thereby changing STIM1 interactions with EB1.

#### 2.1.3 STIM1 variants

Alternative splicing is another important mechanism for regulating protein function and STIM1L was the first STIM1 splice variant identified (Darbellay et al., 2011). Alternative splicing on exon 11 results in the insertion of 106 residues between the SOAR/CAD and P/S region in the C-terminal region of STIM1, which functions as an actin binding domain (ABD) (Figure 1). In contrast to the ubiquitous expression of STIM1, STIM1L appears to be restricted to striated muscle and brain in rodents (Darbellay et al., 2011) and skeletal muscle in humans (Horinouchi et al., 2012), although it is found in neonatal rat cardiomyocytes and in adult rodent hearts under stress (Luo et al., 2012; Sabourin et al., 2018). In skeletal muscle, the actin binding domain in STIM1L enables it to form permanent clusters with Orai1 thereby allowing for immediate activation of SOCE, which may be critical in excitable cells where there are large rapid changes in ER and cytosolic Ca^2+^ levels facilitating faster and more efficient refilling of ER (Darbellay et al., 2011). Database analysis predicts that several other STIM1 splice variants may occur and to date, two variants STIM1A and STIM1B have been characterized (Knapp et al., 2021; Ramesh et al., 2021). STIM1A contains an additional A domain comprising 31 residues, adjacent to the ID domain (Figure 1) and is highly conserved from fish to birds to mammals. STIM1A was found in heart, kidney, astrocytes, and testes, but was not present in T-cells. STIM1 and STIM1A both co-localized with Orai1 after ER Ca^2+^ depletion; surprisingly, however, STIM1A appears to function in a dominant negative manner, resulting in a decrease in SOCE possibly by interfering with the interaction between the STIM1 CAD/SOAR domain and Orai1 (Knapp et al., 2021). STIM1B has a truncated C-terminus that includes a novel B domain downstream of the ID domain (Ramesh et al., 2021). STIM1B was reported to be exclusively found in the brain and compared to STIM1 exhibits slower formation of oligomers in response to store depletion and differential interactions with all 3 Orai isoforms. The altered function of STIM1B appears to be primarily linked to the new B-domain rather that the absence of P/S, EB, and PB domains (Ramesh et al., 2021).

### 2.2 STIM2

In contrast to invertebrates that have a single STIM gene, mammals have two genes, STIM1 and STIM2. The STIM2 gene was cloned in 2001 and its fundamental structure characterized (Williams et al., 2001); however, its function was unknown.

#### 2.2.1 STIM2 structure

In contrast to STIM1 where a variable fraction is located at the plasma membrane, a di-lysine ER-retention signal restricts STIM2 to the ER (Ercan et al., 2012). The overall structure of STIM2 is similar to STIM1 particularly in the N-terminal ER region (Figure 1), which contains SP, cEF, hEF, and SAM domains. The unusually long 101 residue STIM2 SP appears to contribute to reduced ER localization leading to a pool of uncleaved cytosolic preSTIM2 (Graham et al., 2011). It has been reported that the cytosolic preSTIM2 interacts with Orai1 at the plasma membrane potentially regulating basal Ca^2+^ levels (Graham et al., 2011). In addition, a 91aa fragment of the STIM2 signal peptide (SPF) is also released into the cytosol and may regulate NF-κB transcription (Graham et al., 2011). The rest of the luminal STIM2 N-terminus shares >80% homology with STIM1 (Stathopulos et al., 2009); however, differences in only 3 amino acids in the cEF hand sequence results in a 2-fold lower affinity for Ca^2+^ than STIM1 making it more sensitive to small changes in ER Ca^2+^ concentrations (Zheng et al., 2011). Despite a high degree of similarities between the SAM domains for STIM1 and STIM2, subtle differences in the STIM2 SAM domain result in a substantial increase in its stability (Zheng et al., 2008), which attenuates its rate of oligomerization in response to ER Ca^2+^ depletion (Zheng et al., 2011). In addition, small differences in the STIM2 TM domain compared to STIM1-TM also slows the transduction of ER Ca^2+^ depletion signal to the cytosolic portion of the protein (Zheng et al., 2018).

The C-terminal cytosolic region of STIM2 contains similar CC1, OASF, SOAR/CAD, ID, and terminal PB domains to those found in STIM1 (Grabmayr et al., 2020). There is exceptional homology between the STIM1 and STIM2 SOAR/CAD sequences; however, the switch of a single phenylalanine in STIM1 SOAR/CAD to leucine in STIM2 markedly reduces its ability to open Orai1 channels (Wang et al., 2014). Small differences in the S1α helix in the STIM2 CAD/SOAR domain compared to the STIM1 domain weakened the interactions between the CC1 and CC3 domains of STIM2. This resulted in a more open conformation of the STIM2 CAD/SOAR region resulting in increased clustering in ER-PM junctions under resting conditions (Subedi et al., 2018; Zheng et al., 2018). Moreover, the STIM2 PM domain has higher affinity for phosphatidylinositol biphosphate (PIP_2_) than STIM1, which also helps facilitate STIM2 clustering with minimal changes in ER Ca^2+^ levels (Bhardwaj et al., 2013). On the other hand, these changes in the STIM2 CAD/SOAR domain reduced its activation of Orai1 compared to STIM1 (Zheng et al., 2018). STIM2 also contains a proline/histidine (P/H) rich region instead of the P/S region found in STIM1. While an EB domain in STIM2 has not been conclusively identified, in neurons STIM2 has been shown to bind EB3 via a similar TRIP motif to that seen in STIM1 (Pchitskaya et al., 2017). There is also a calmodulin binding site close to the PB domain (Bauer et al., 2008).

#### 2.2.2 STIM2 regulation

STIM2 like STIM1 has numerous phosphorylation sites (>30), most of which are in the cytosolic C-terminal region (Hornbeck et al., 2004); however, little is known about their function or which kinases are involved. Like STIM1, STIM2 has cysteine residues in its luminal domain; two of them Cys53, and Cys60 are conserved with STIM1, and one Cys15 is unique to STIM2. All three residues can be S-nitrosylated leading to a synergistic stabilization of the EF-SAM region, reduced basal cytosolic Ca^2+^ and lower STIM2-mediated SOCE (Novello et al., 2020). In contrast to STIM1, STIM2 constitutively clusters at the ER-PM junctions in both mobile and immobile clusters with changes in both IP_3_R function and ER Ca^2+^ levels being the driving factors contributing to the increases or decreases of immobile clusters of STIM2 in ER-PM junctions (Ahmad et al., 2022). Under basal conditions the STIM2/Orai1 complex regulates basal Ca^2+^ concentrations whereas following agonist stimulation STIM1 forms clusters with STIM2 in response to a decrease in ER Ca^2+^ combined with a close association with IP_3_R. Collectively these findings suggest that immobilization of STIM2 clusters is an early response to decreased ER Ca^2+^ levels, which is facilitated by IP_3_R in the region of STIM2 clusters and acts as a “checkpoint” for Ca^2+^ entry (Ahmad et al., 2022).

#### 2.2.3 STIM2 variants

In 2015, there were two reports describing a novel STIM2 splice variant, STIM2β (also referred to as STIM2.1), which antagonized STIM1-Orai1 mediated SOCE (Miederer et al., 2015; Rana et al., 2015). In different cell/tissue types there is a wide range in the expression ratio of STIM2.1 to the original STIM2 variant now known as STIM2.2 (or STIM2α). STIM2.1 also blunted the STIM2.2-mediated SOCE (Miederer et al., 2015). The antagonistic effects of STIM2.1 and wide range of cell-dependent ratios of STIM2.1/STIM2.2, might in part, account for the different conclusions of the earlier studies on STIM2 function. STIM2.2 is characterized by an 8 amino acid insertion in SOAR domain of STIM2.1; however, the mechanism by which this leads to SOCE inhibition remains unclear. It is possible that STIM2.1 forms heterodimers with STIM1 or STIM2.2, thereby preventing them from binding to Orai1, or STIM2.1 could actively inhibit SOCE via direct interaction with Orai1 (Rana et al., 2015). It is worth noting that bioinformatics analysis predicts at least an additional 4 human STIM2 splice variants, although to date, only STIM2.1 and STIM2.2 have been identified (Berna-Erro et al., 2017), suggesting that there is still much left to discover regarding STIM2 and its variants.

## 3 Orais—Gene and Protein Structures

### 3.1 Orai1

In 2001, Rao and colleagues identified major Ca^2+^ signaling defects in T-cells from a patient with severe combined immunodeficiency (SCID) (Feske et al., 2001). Subsequent studies with these cells demonstrated that although SOCE was almost completely abolished, STIM1 levels were normal (Feske et al., 2005), illustrating that while STIM1 was essential for SOCE it did not act alone. The fact that STIM1 was primarily localized to the ER strongly suggested that an unidentified plasma membrane Ca^2+^ channel was also involved in activating SOCE. Using genetic linkage analysis of the SCID patients and their family combined with a high throughput siRNA screen of SOCE in *Drosophila* S2 cells, a novel protein they named Orai1 and two human homologues Orai2 and Orai3 were identified (Feske et al., 2006); a single point mutation in Orai1 was responsible for the defective SOCE in cells from the SCID patients (*The name Orai originates from Greek mythology where Orai are the keepers or guardians of the gates of heaven (* *Feske et al., 2006* *)*). Two additional studies published in 2006 confirmed the essential role of Orai1 in SOCE and correctly predicted that it had 4 transmembrane domains with both C- and N-terminal regions in the cytosol (Vig et al., 2006b; Zhang et al., 2006). While Orai1 was clearly essential for SOCE, it had no homology with any other ion channel. As a result, initially it was unclear whether Orai1 was the elusive SOCE channel or instead a regulator of the channel (Shuttleworth, 2012). However, subsequent studies quickly established that interactions between STIM1 and Orai1 were required for SOCE and that Orai1 itself formed the plasma membrane channel that allows for Ca^2+^ entry to occur (Mercer et al., 2006; Prakriya et al., 2006; Soboloff et al., 2006; Yeromin et al., 2006).

#### 3.1.1 Orai1 structure

**FIGURE 2:**
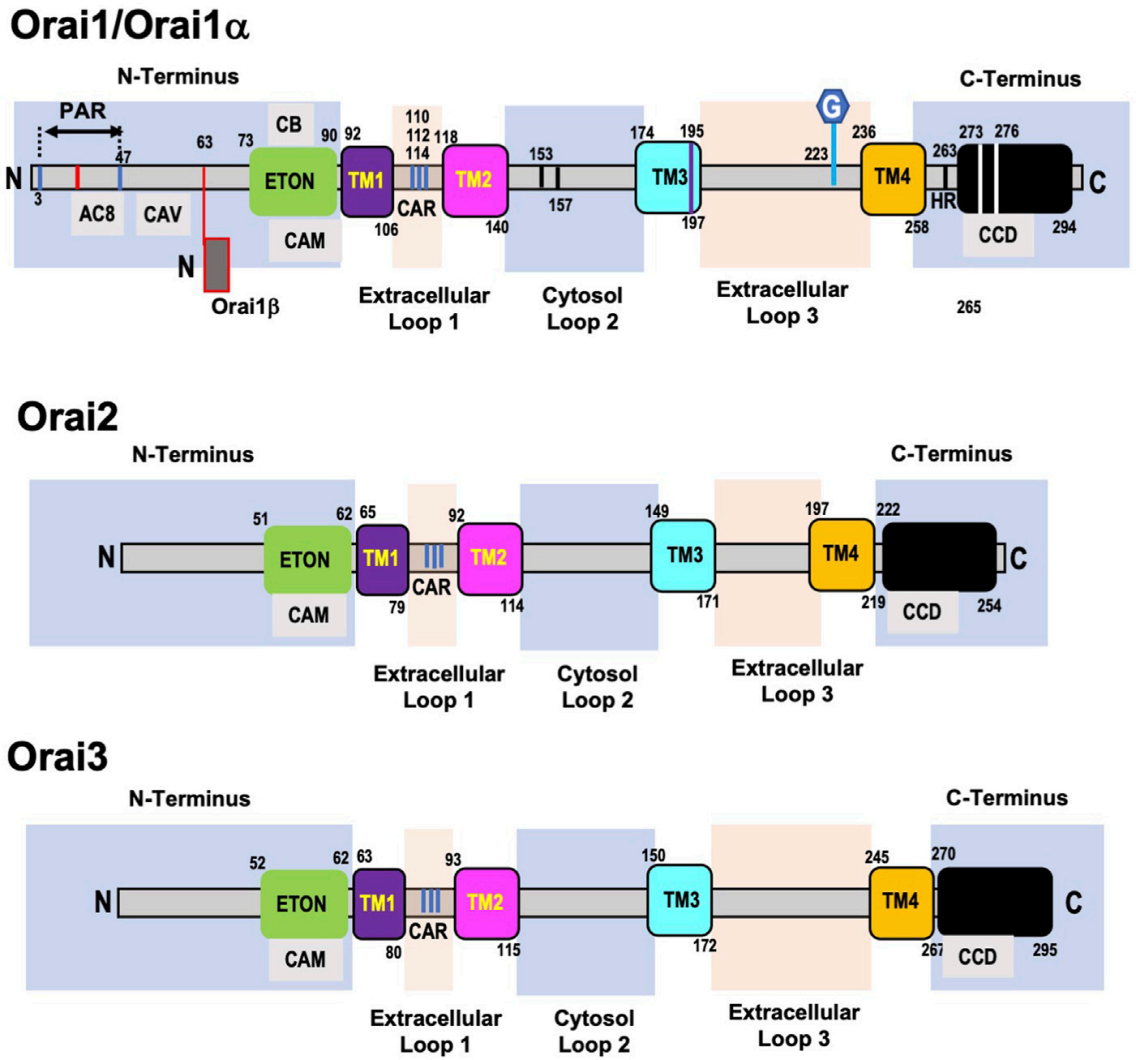
Domain structure of human ORAI1, ORAI2, and ORAI3 proteins. Orai1 (Orai1α) has a proline arginine region (PAR) close to the end of the N-terminal domain, which includes an interacting site for adenylate cyclase-8 (AC8). This is followed by a caveolin binding region (CAV) and adjacent to the plasma membrane is the Extended Transmembrane Orai1 N-terminal (ETON) region, which contains calmodulin (CAM) and cholesterol binding (CB) domains. Following the first transmembrane domain (TM1) is the first extracellular loop, which includes a Ca^2+^ accumulating region (CAR). A region in the intracellular loop 2—153–157—is thought to regulate Ca^2+^ dependent inactivation of Orai1 and interaction between loop2 and the ETON region regulate channel activation. A cysteine residue (Cys195) at the end of the third transmembrane domain (TM3) has been implicated in the redox regulation of Orai1 function. The second extracellular loop (loop3) contains a N-glycosylation site at N223, of unknown function. The cytosolic C-terminus of Orai1 is connected to the 4th transmembrane domain (TM4) via a highly conserved hinge region (HR) and contains highly conserved coiled-coil domain (CCD). The C-terminal region essential for recruiting STIM1 and Orai1 channel activation. Alternative translation initiation results in Orai1β which lacks the first 63 amino acids of Orai1/Orai1α. The architecture of Orai2 and Orai3 are very similar to Orai1 with a few key differences. Orai2 and Orai3 have a truncated N-terminal region which lacks the PAR present in Orai1. They also lack an 18aa region in the N-terminus which regulates the slow reactivation that follows fast CDI in Orai1. Similar to Orai1 both Orai2 and Orai3 contain ETON region adjacent to the TM1, which includes a CAM domain. The TM1 domains are almost completely conserved, and the other 3 transmembrane domains exhibit a high level of homology across all Orai isoforms. However, Orai2 and 3 lack the cysteine residue in TM3 and the N-glycosylated site on extracellular loop 3 which are present in Orai1. Orai3 has a much longer loop 3 compared to both Orai1 and Orai2, but like Orai1, Orai2, and Orai3 also have coiled-coil domains their C-terminal regions.

The domain structure of Orai1 (Figure 2) consists of four transmembrane (TM) helices connected by two extracellular loops and one intracellular loop, with both the N- and C-terminal regions located in the cytosol. There is a proline arginine (PA) region close to the end of the N-terminal region that is involved in Orai1 reactivation (Frischauf et al., 2011), and includes an interacting site for adenylate cyclase-8 (AC8) (Willoughby et al., 2012). PIP_2_ binding in the same region as AC8 has been reported to enhance Orai1-STIM1 interactions (residues 28–33) (Calloway et al., 2011). Adjacent to the plasma membrane is an α-helical extension of the TM1 domain known as the Extended Transmembrane Orai1 N-terminal (ETON) region. Almost the entire ETON region has been reported to be essential for binding with STIM1 and allowing STIM1-dependent Ca^2+^ entry (Derler et al., 2013); however, others have suggested that STIM1 interaction with the ETON region is not necessary for channel activation (Fahrner et al., 2018a). The ETON region contains calmodulin and cholesterol binding domains. The calmodulin binding domain has been reported to play a role in Ca^2+^ dependent inhibition (CDI) of SOCE (Mullins et al., 2009; Kar et al., 2014); however, it has also been suggested that this region is involved in CDI-independent of calmodulin binding (Mullins et al., 2016).

The interaction of cholesterol with Orai1 is complex with reports that it inhibits its activity and decreases SOCE (Derler et al., 2016b) on the other hand decreasing cholesterol reduced SOCE due to increased internalization of Orai1 channels (Bohorquez-Hernandez et al., 2017). Derler *et al.*, found that cholesterol depletion increased SOCE and identified a cholesterol binding motif in the region of the ETON domain that interacts with calmodulin (Derler et al., 2016b). Others have reported that cholesterol depletion reduces SOCE via increased internalization of Orai1 (Bohorquez-Hernandez et al., 2017). The role of cholesterol on SOCE is complicated by the fact that the SOAR region of STIM1 also has a cholesterol binding site (Pacheco et al., 2016). A caveolin binding domain has also been identified in the N-terminus and caveolin binding to Orai1 has been reported to increase SOCE (Yeh and Parekh, 2015; Bohorquez-Hernandez et al., 2017). However, mutation of these residues did not prevent the enhancement of SOCE that occurs in the presence of caveolin suggesting that Orai1 may contain another caveolin binding domain (Yeh and Parekh, 2015).

The first extracellular loop (loop 1) contains a Ca^2+^ accumulating region (CAR), formed by aspartate residues, which increase local Ca^2+^ concentrations facilitating Ca^2+^ entry even when extracellular Ca^2+^ concentrations are low (Frischauf et al., 2015). The TM2 and TM3 domains, are connected via an intracellular loop (loop2) and both have short helical extensions in the cytosol. Loop2 has been implicated in regulating fast CDI of the Orai1 channel possibly via blocking Ca^2+^ entry (Srikanth et al., 2010) and interactions between loop2 and the ETON region have been shown to regulate channel activation (Fahrner et al., 2018a). A cysteine residue was identified at the end of the TM3 domain, close to extracellular loop3, which has been implicated in the redox regulation of Orai1 function (Bogeski et al., 2010; Alansary et al., 2016). The second extracellular loop (loop3) that connects TM3-TM4 was shown to interact with loop1, potentially fine tuning Ca^2+^ accumulation in the CAR (Frischauf et al., 2015). Loop3 of Orai1 also contains a distinct N-glycosylation site at N223; the function of this modification is not known, but in some cell types, loss of this modification resulted in an increase in Ca^2+^ entry, suggesting that it may play a cell-specific role in regulating SOCE (Dorr et al., 2016). TM4 is connected to the cytosolic C-terminus of Orai1 via a highly conserved hinge region (Hou et al., 2012; Fahrner et al., 2018b). Residues in the C-terminal of Orai1 are essential for recruiting STIM1 and channel activation (Mcnally et al., 2013).

Each of the transmembrane domains contribute to the regulation of the Orai1 channel as indicated by the numerous gain and a loss of function mutations that have been identified throughout the regions (Yeung et al., 2018; Tiffner et al., 2020). Mutations of Gly98 and Val102 in the TM1 domain led to constitutively active, non-selective currents, indicating they contribute to keeping the channel closed (Zhang et al., 2011; Mcnally et al., 2012). Multiple mutations in TM2 also result in constitutively active Ca^2+^ channels and are associated with various diseases including cancer (Endo et al., 2015; Frischauf et al., 2017). The TM3 domain contributes to Orai1 channel gating and ion selectivity as shown by the effects of mutations of Trp176 and Gly183 (Srikanth et al., 2011). The mutation of Pro245 in the TM4 domain to lysine still required STIM1 for activation of Orai1 but resulted in very slow inactivation of the channel and is associated with a myopathy in humans (Nesin et al., 2014). A key feature of the Oria1 channel is its high selectivity for Ca^2+^, and this has been shown to be due to a set of conserved amino acids, Glu106, Glu190, Asp110, Asp112, Asp114 in TM1 and TM3 and the extracellular loop 1 (Prakriya et al., 2006; Vig et al., 2006a; Yeromin et al., 2006; Yamashita et al., 2007). As noted above mutation of Val102 in the TM1 also contributes to the Ca^2+^ selectivity of the Orai1 channel (Mcnally et al., 2012).

#### 3.1.2 Orai1 regulation

Six phosphorylation sites have been identified in the N-terminal region and four in the C-terminal region of Orai1 (Hornbeck et al., 2004). Ser27 and Ser29 have both been shown to be phosphorylated by PKCβ (Kawasaki et al., 2010); Ser34 is a target of PKG (Wang et al., 2015) and PKA (Zhang et al., 2019). In each case phosphorylation either directly inhibits SOCE or contributes to CDI. The kinases and function of other phosphorylation sites have yet to be identified (Hornbeck et al., 2004). Alternative translation initiation results in a long variant Orai1α, which is the full length Orai1 and a short variant Orai1β adding another layer of functional regulation (Fukushima et al., 2012). Orai1β lacks the first 63 amino acids of Orai1α, a region that as noted above contains several potentially important signaling regions (Putney, 2018). Orai1α exhibited substantially slower plasma membrane mobility compared to Orai1β possibly as a result of the absence of the caveolin and PIP_2_ binding regions; nevertheless, both isoforms form puncta with STIM1 and facilitated SOCE (Fukushima et al., 2012). While Orai1α and Orai1β appear to be functionally indistinguishable regarding SOCE, Orai1α exhibited stronger CDI (Desai et al., 2015). Interestingly, only Orai1α participated in the non-store-dependent arachidonic acid regulated Ca^2+^ (ARC) channels, suggesting the possibility of physiologically distinct roles for the two isoforms (Desai et al., 2015).

### 3.2 Orai2 and Orai3

Feske *et al.*, identified Orai2 and Orai3 during their initial studies characterizing the role of Orai1 in SOCE (Feske et al., 2006). Phylogenetic analysis showed that while Orai1 and Orai2 were present in vertebrates, Orai3 was only observed in mammals, suggesting that Orai3 evolved from Orai1 not Orai2 (Cai, 2007).

#### 3.2.1 Orai2 and Orai3 structure

Like Orai1, Orai2, and Orai3 are ubiquitously expressed (Hoth and Niemeyer, 2013). The TM1 domains are almost completely conserved and the other 3 transmembrane domains exhibit a high level of homology across all Orai isoforms (Hoth and Niemeyer, 2013). Both Orai2 and Orai3 have a truncated N-terminal region which lacks the PA region seen in Orai1 (Shuttleworth, 2012). They also lack an 18aa region in the N-terminus with contributes to regulation of slow reactivation that follows fast CDI in Orai1 (Frischauf et al., 2011). This is consistent with the observation that Orai2 and Orai3 both exhibit fast CDI but this is followed by a slower inhibitory phase rather that the reactivation observed in Orai1 (Lis et al., 2007). There is a highly conserved region of 22 amino acids immediately before TM1, which includes the CAM binding domain (Shuttleworth, 2012). Despite 75% homology between cytosolic loop2 of Orai3 and Orai1, differences are sufficient to eliminate the role of the N-terminal domain in channel activation (Fahrner et al., 2018a). Orai2 and 3 lack the cysteine residue in TM3 that occurs in Orai1, resulting in decreased sensitivity of Orai2 and 3 to redox stress (Bogeski et al., 2010). Unlike Orai1, Orai2 and 3 are not N-glycosylated on extracellular loop 3; moreover, Orai3 has a much longer loop 3 compared to both Orai1 and Orai2, although the functional consequence of this is not known (Frischauf et al., 2011; Shuttleworth, 2012). Like Orai1, Orai2 and 3 also have coiled-coil domains in their C-terminal regions. Differences in binding affinities for STIM1 in the Orai C-terminal regions is reflected by the extent to which they trigger SOCE when overexpressed with STIM1, with Orai1 exhibiting larger SOCE compared to either Orai2 or Orai3.

#### 3.2.2 Orai2 and Orai3 variants

There are two murine Orai2 splice variants with one Orai2S lacking 14 N terminal amino acids of Orai2L, with Orai2S potentially acting in a dominant negative fashion to block STIM1/Orai1 SOCE (Gross et al., 2007); to date this has not been observed in humans.

## 4 STIM and Orai Mediated Ca^2+^ Signaling

SOCE is characterized by a very specific Ca^2+^ current, *I*_*crac*_, which reflects key biophysical properties including very high specificity for Ca^2+^. STIM and Orai proteins are also involved in less selective store operated Ca^2+^ channels, resulting in a Ca^2+^ current known as *I*_*soc*_, which can involve interactions of STIM and Orai proteins with transient receptor potential (TRP) channels (Ong and Ambudkar, 2011). While STIM and Orai proteins are essential for SOCE (*I*_*crac*_), it has been shown that TRP channel mediated Ca^2+^ entry is not dependent on either STIM or Orai proteins (Dehaven et al., 2009). Therefore, the discussion below of STIM and Orai mediated Ca^2+^ signaling will not include consideration of TRP channels, which are reviewed in detail elsewhere (Vazquez et al., 2004; Chen X. et al., 2020). There is however, a store-independent Ca^2+^ channel, that is activated by arachidonic acid (AA) or its metabolite leukotriene and is dependent on both STIM and Orai proteins (Zhang et al., 2018). This channel commonly known as arachidonate-regulated Ca^2+^ (ARC) channel is responsible for a highly selective Ca^2+^ current, *I*_*arc*_, which has distinct physiological roles from SOCE (Zhang et al., 2018). Considering the essential role of STIM and Orai proteins in ARC channel activity, this is also discussed below.

### 4.1 Store Operated Ca^2+^ Entry

As noted above, STIM1 and Orai1 are essential for SOCE and required for *I*_*crac*_; therefore, we will focus initially on the canonical function of STIM1 and Orai1 in the regulation of SOCE. The potential roles of STIM2, Orai2, and Orai3 will be considered later.

Under basal resting conditions, the cEF hand of STIM1 is bound to Ca^2+^ and STIM1 is distributed diffusely in the ER membrane. The cytosolic CAD/SOAR region, via interactions with CC1 domain, is locked in an inactive conformation close to the ER/SR membrane. When ER/SR Ca^2+^ levels decrease, Ca^2+^ dissociates from the cEF hand initiating a conformational change in the hEF and SAM domains, which begins the formation of STIM1 oligomers. This conformational change within the ER/SR lumen is transmitted to the cytosol via the STIM1 TM domain, resulting in a release of the SOAR/CAD region. Subsequent conformational changes of all three CC domains enhances STIM1 oligomerization, exposes the SOAD/CAD region to facilitate binding to Orai1 as well as extending the C-terminal region towards the plasma membrane (Derler et al., 2016a; Lewis, 2020). Under resting conditions, STIM1 diffuses freely in the ER membrane, whereas Orai1 diffusion is somewhat constrained possibly due to binding with other proteins (Wu et al., 2014) or the formation of supra-molecular Orai1 clusters (Peckys et al., 2021). Once activated the extended STIM1 region is trapped at ER-PM junctions via interactions of the PB domain with the plasma membrane, facilitated in part by PIP_2_. Subsequently, STIM1 traps Orai1 via binding of the SOAR/CAD region to the Orai1 C-terminal region (Wu et al., 2014). The trafficking chaperone, uncoordinated 93 homolog B1 (UNC93B1), has been reported to play an important role in the early activation of STIM1, facilitating its extension. This appears to result in a more efficient interaction between STIM1 and Orai1 channels. However, UNC93B1 does not play a role in the translocation of STIM1 to the plasma membrane or in gating of the Orai1 channel (Wang and Demaurex, 2022).

Key regions of C-terminal domains of both STIM1 and Orai1 form a STIM1-Orai1 association pocket (SOAP) and mutations in this region prevent STIM1 activation of Orai1 (Derler et al., 2016a). The Ca^2+^ channel itself is composed of hexameric Orai1 subunits arranged around a pore created by TM1 domains that extend across the membrane and into the cytosol (Hou et al., 2012; Hou et al., 2020). The precise mechanism by which binding of STIM1 to the Orai1 C-terminal region leads to opening of the Ca^2+^ channel, remains uncertain. However, it has been proposed that binding of the SOAR/CAD region of STIM1 to Orai1 results in a conformational change in the hinge region of the cytosolic extension of TM4. This results in conformational changes in TM4 itself, disrupting interactions with TM3 followed by further conformational changes in TM3/TM2 leading to rotation of the TM1 helices and subsequent channel activation (Zhou et al., 2017). While the N-terminal region of Orai1 is essential for channel activation, it is unclear whether this involves interaction with STIM1. It has been suggested that the N-terminus might regulate channel activity via interactions with other domains such as TM3 or cytosolic loop2 (Fahrner et al., 2018b). It is important to note that while STIM1 and Orai1 are essential for SOCE, there are a growing number of accessory proteins that have been identified as regulating SOCE, which are reviewed in detail elsewhere (Srikanth and Gwack, 2012; Woo et al., 2018; Berlansky et al., 2021).

Most Ca^2+^ channels are regulated by feedback inhibition by Ca^2+^, a process known as CDI; this is also true for Orai1-mediated SOCE. As noted earlier, STIM1 contains an inhibitory domain (ID_STIM_) that is essential for CDI; surprisingly however, it is does not appear to be the primary Ca^2+^ sensor responsible for initiating CDI. Calmodulin, which binds to the N-terminal of Orai1, was thought to be the CDI sensor, but this turned out not to be the case (Lewis, 2020). Mullins *et al.*, found that two residues in the Orai1 pore, Trp76 and Tyr80 played a key role in CDI leading to conformation changes, which inactivated the channel (Mullins et al., 2016). Subsequently, they found that ID_STIM_ binding to Trp76 was required for full CDI (Mullins and Lewis, 2016). The Orai1β splice variant did not exhibit CDI indicating that the first 63 amino acids of Orai1 that are absent in Orai1β, contributed to CDI (Zhang et al., 2019). There are AC8 and caveolin binding domains in that 63 amino acid region, which have been shown to be essential for CDI (Zhang et al., 2019). Based on these findings a model was proposed where cAMP generated by Ca^2+^ dependent AC8 resulted in phosphorylation of Ser34 of Orai1 by protein kinase A (PKA), which induced CDI (Zhang et al., 2019). How Ser34 phosphorylation regulates CDI remains to be determined, although it was speculated that it may facilitate binding of ID_STIM1_ to Orai1 (Zhang et al., 2019). However, concern has been raised regarding the generalization of this mechanism due to the limited tissue distribution of AC8 (Hofer, 2019). Interestingly, compared to Orai1, Orai2, and Orai3 exhibit faster CDI, which is mediated by three conserved glutamates in their C-terminal domains (Lee et al., 2009).

A consequence of the emphasis on STIM1 and Orai1 in understanding the molecular mechanisms underlying SOCE is that our understanding of the potential roles of STIM2, Orai2, and Orai3 has been neglected. Early studies showed that overexpression of Orai2 and Orai3 with STIM1 resulted in SOCE and generation of *I*_*crac*_ albeit with some differences in their biophysical characteristics compared to Orai1 (Mercer et al., 2006; Dehaven et al., 2007; Lis et al., 2007). However, the physiological role of Orai2 and Orai3 in regulating physiological Ca^2+^ signaling remained unclear. The role of STIM2 is also not well understood. Early studies reported contradictory findings regarding STIM2 function, with some reports suggesting that it facilitated SOCE in a similar manner to STIM1, whereas others indicated that STIM2 inhibited the actions of STIM1. STIM2 was found to form pre-made clusters with Orai1 and it was believed that this played an important role in regulating basal cytosolic and ER Ca^2+^ levels (Brandman et al., 2007). It has also been suggested that STIM2 might act as an adaptor protein regulating STIM1 function (Berna-Erro et al., 2017).

It has been proposed that the difficulty in identifying clear roles for Orai2/3 and STIM2 is because the protocols used to generate maximal SOCE signals and *I*_*crac*_ currents do not represent normal physiological stimuli for Ca^2+^ signaling, thereby hiding potentially more subtle roles for these proteins (Yoast et al., 2020; Emrich et al., 2021). Studies by Trebak and colleagues suggest that under more physiological conditions, Orai2 and Orai3 form heteromultimers with Orai1, attenuating its activity, resulting in a larger bandwidth of Ca^2+^ signals (Yoast et al., 2020). Moreover, they have also proposed that physiological Ca^2+^ signaling requires STIM1 and STIM2 interactions to further finetune intracellular Ca^2+^ signaling (Emrich et al., 2021). While the concept that all five STIM/Orai isoforms work together to regulate the Ca^2+^ signaling responses to agonist stimulation clearly complicates the understanding of the function of individual proteins, it also represents a potentially elegant solution for the diverse roles of SOCE channels. Such a model would allow for Ca^2+^ signaling to be fine-tuned due to cell/tissue specific differences in expression of these five proteins. Clearly, a great deal of additional work is needed to determine how the five STIM/Orai isoforms work together under physiological conditions and whether alterations in stoichiometry could account for the diverse functions of SOCE in different tissues and cells. Understanding how different STIM and Orai variants fit in with this model also remains to be determined.

### 4.2 Store Independent Ca^2+^ Entry

For many years SOCE was widely considered to be the primary agonist-mediated Ca^2+^ signaling pathway, but in 1996 Shuttlesworth and Thompson identified a plasma membrane Ca^2+^ entry pathway that was independent of intracellular Ca^2+^ stores (Shuttleworth and Thompson, 1996). In a series of studies, they identified arachidonic acid as the agonist responsible and named the resulting Ca^2+^ current *I*_ARC_ (for arachidonate-regulated calcium current) (Shuttleworth, 1996; Shuttleworth and Thompson, 1998; Mignen and Shuttleworth, 2000). Several different agonists were subsequently shown to activate a store-independent, arachidonic acid (AA) dependent Ca^2+^ entry pathway in several cell types (Munaron et al., 1997; Broad et al., 1999; Guibert et al., 2004); however, the identity of the channel proteins remained elusive (Shuttleworth et al., 2004). While the focus on STIM1 had been its role as the ER/SR Ca^2+^ sensor regulating SOCE, it had originally been identified as a plasma membrane protein (Manji et al., 2000; Williams et al., 2001; Williams et al., 2002); consequently, Mignen *et al.*, examined whether it also played a role in ARC mediated Ca^2+^ entry (Mignen et al., 2007). They demonstrated that ARC channels were regulated by the plasma membrane pool of STIM1, with its N-terminal domain in the extracellular environment (Mignen et al., 2007). In subsequent studies they found that Orai1 and Orai3, but not Orai2 were also required for ARC channel activity (Mignen et al., 2008; 2009).

Activation of an Orai1/Orai3 Ca^2+^ channel by leukotrieneC_4_ (LTC_4_) that was also STIM1-dependent was reported to have very similar biophysical characteristics as the ARC channel (Gonzalez-Cobos et al., 2013; Zhang et al., 2013). However, in contrast to ARC channel activation ER/SR STIM1 rather than plasma membrane STIM1 was found to be sufficient for LTC_4_ regulated Ca^2+^ (LRC) channel activation (Zhang et al., 2013). There was no formation of STIM1 puncta in response to LTC_4_, but the interaction between the STIM1 CC domains and Orai3 was necessary for channel activity (Zhang et al., 2013). It was subsequently shown that the biophysical characteristics of LTC_4_ and ARC channel activation were identical requiring both Orai1 and Orai3, and that metabolism of AA to LTC_4_ was necessary for full activation of the channels (Zhang X. et al., 2014).

It has been suggested that the apparent differences in the pools of STIM1 required for channel activation by AA and LTC_4_ was dependent on whether patch clamped cells or intact cells were studied and that ER/SR STIM1 was sufficient for ARC activation in intact cells (Zhang X. et al., 2014). However, the precise role of STIM1 in the regulation of ARC channels remains unclear because some studies have shown that while Orai1 and Orai3 are essential for ARC activation, STIM1 may not be required (Dubois et al., 2014; Goswamee et al., 2018). On the other hand, Thompson and Shuttlesworth reported that PKA-mediated phosphorylation of Thr389 of the cytosolic domain of plasma membrane STIM1 was necessary for ARC channel activation (Thompson and Shuttleworth, 2015). Thus, while there appears to be a consensus that Orai1 and Orai3 are essential components of ARC/LRC channels, the role and cellular pool of STIM1 remains an open question (Zhang et al., 2018). It is has also not been settled whether AA and LTC_4_ activate the channels independently or if metabolism of AA to LTC_4_ is required (Zhang et al., 2018).

## 5 Metabolic and Mitochondrial Roles of STIM and Orai

The metabolic roles of STIM and Orai have been described in immune cell populations (Vaeth et al., 2017); however, the role of these proteins in regulating metabolism and mitochondrial function has been less studied in other organs and cell types. In this section, we will discuss the contribution of STIM and Orai isoforms to the regulation of glucose and lipid metabolism and mitochondrial function in various non-immune cells of different organs, including cardiomyocytes, hepatocytes, and skeletal muscle cells.

Plenty of evidence points in the direction of STIM and Orai proteins regulating fatty acid and lipid metabolism. A recent study from Maus *et al.*, (Maus et al., 2017), showed that cells lacking either STIM1 or Orai1 had reduced SOCE, which mediated significantly high levels of lipid droplet deposition and increased lipophagy, and was shown in numerous organs including the liver, heart, and skeletal muscle. Consistent with that study, we showed that hearts from cardiomyocyte-specific STIM1-KO mice had lipid droplet accumulation, triglyceride accumulation, and altered expression of several fatty acid metabolism proteins (Collins et al., 2019). We also found reductions in insulin-mediated cardiac protein kinase b (Akt) activation, which has been shown to occur in other STIM1 cardiomyocyte knockdown models. Other studies have shown that activation of STIM1/Orai1-mediated SOCE activated Akt, glycogen synthase kinase 3 beta (GSK3β) and mTORC2 signaling whereas pharmacological inhibition attenuated this pathway (Benard et al., 2016), providing further support for the key role of STIM1 in the regulation of metabolism.

In addition to impaired cardiac glucose metabolism in cardiomyocyte-specific STIM1-KO mice, we also showed significant changes in mitochondrial size and shape as well as evidence of increased mitochondrial fission through reductions in Mitofusin (Mfn2) and increased Dynamin related protein 1 (Drp-1) expression (Collins et al., 2014; Collins et al., 2019). Mitochondrial structural abnormalities have been identified in virtually all STIM and Orai KO and overexpression models in various cell types. For example, skeletal muscle overexpression of STIM1 has been shown to have a dystrophic-like phenotype associated with the presence of swollen mitochondria (Goonasekera et al., 2014). Henke *et al.*, (Henke et al., 2012), also showed that fibroblasts lacking either STIM1 or Orai1 were more susceptible to oxidative stress and showed that the mitochondria from STIM1-KO cells were abnormally shaped with abnormal cristae, had increased Ca^2+^ load, increased glutathione levels, and there was a significant increase in transcription of antioxidant genes, suggesting that STIM1 is an important regulator of mitochondrial function. The same group also showed that oxidative stress reduced SOCE in hippocampal neurons and that knockdown of Orai1 was protective against glutathione depletion (Henke et al., 2013). In support of these studies, several of the cardiomyocyte-specific KO and overexpression models of STIM and Orai proteins show significant mitochondrial structural abnormalities correlating with reductions in mitochondrial function and alternations in mitochondrial quality control (Collins et al., 2014; Correll et al., 2015; Collins et al., 2019; Segin et al., 2020; Gammons et al., 2021). Silva-Rojas *et al.* examined gain of function mutations in both STIM1 and Orai1 and found that this resulted in increased SOCE and promoted abnormal Ca^2+^ handling and mitochondrial activity. Specifically, the authors used mice with mutant STIM1, STIM1^R304W/+^ mice, and found that the abnormal Ca^2+^ handling was the result of changes in the expression of several key proteins including sarco/endoplasmic reticulum Ca^2+^ ATPase (SERCA) and ryanodine receptor (RyR). The abnormal mitochondrial activity was the result of changes in the expression of several mitochondrial proteins which include mitochondrial transcription factor A (Tfam), peroxisome proliferator-activated receptor gamma coactivator (PGC1α), nuclear respiratory factor 1 (Nrf1), Sirtuin 1 (Sirt1), and mitochondrial fission 1 protein (Fis1) and an increase in apoptosis (Silva-Rojas et al., 2021). These studies suggest that STIM1 and Orai play significant roles in modulating mitochondrial function but could also regulate mitochondrial quality control and redox signaling.

It has also been shown that mitochondrial Ca^2+^ uptake is essential for regulating STIM1, Orai1, and SOCE (Naghdi et al., 2010). In addition, it has been shown that the mitochondrial protein, Mfn2 contributes to STIM1 membrane trafficking (Singaravelu et al., 2011) and that knockdown of either the mitochondrial Ca^2+^ uniporter (MCU) or uncoupling protein 2 (UCP2) resulted in slowed STIM1 oligomerization and reduced SOCE (Deak et al., 2014). This relationship appears to be reciprocal since the lack of STIM1, Orai1, and the inositol triphosphate receptor (IP_3_R) in lymphocytes has been shown to not only result in reductions in mitochondrial proteins such as MCU, but also these lymphocytes had altered mitochondrial metabolism dependent on cAMP response element-binding protein (CREB) (Shanmughapriya et al., 2015). Also, the mitochondrial K^ATP^ channel opener, Diazoxide, has been shown to promote upregulation of STIM1 and Orai1 expression (Sampieri et al., 2019) through mechanisms involving increased phosphorylation of ERK1/2 and NFκB (Gavali et al., 2020), which suggests that STIM1 and Orai may contribute to the cardioprotection associated with diazoxide (Katoh et al., 2002; Hausenloy et al., 2004).

The lack of STIM1/Orai1 seems to adversely impact mitochondrial ultrastructure and function in many cell types although this does not appear to be consistent in neuronal cells. For example, in neuron-like PC12 cells it has been shown that siRNA knockdown of STIM1 increased cell viability in response to injury with 1-methyl-4-phenylpyridinium. This was associated with reductions in apoptosis, ROS production, and prevented mitochondrial dysfunction which was believed to be dependent on Homer1a (Li X. et al., 2013). On the other hand, hydroxydopamine-induced injury in PC12 cells was increased following knockdown of STIM1, resulting in increased apoptosis, decreased mitochondrial function, and mitochondrial Ca^2+^ uptake (Li et al., 2014). Interestingly, Rao *et al.*, (Rao et al., 2015), showed that shRNA knockdown of STIM2 in a traumatic brain injury model also improved neuronal survival through the targeting of mitochondrial apoptosis and preservation of mitochondrial function. Overall, these data suggest that lack of STIM and Orai proteins may be beneficial to mitochondrial-dependent cell survival in some cell types although this does not appear to hold true for cardiomyocytes.

STIM1 has been described as a “metabolic checkpoint” for tumor growth and metastasis in hepatocytes. For example, reductions in STIM1 in hepatocytes mediated a switch from glycolysis to AMPK-mediated fatty acid oxidation (Zhao et al., 2020). It has also been shown that hepatocytes from obese mice have reduced SOCE, occurring due to a reduction in STIM1 translocation, and these changes were associated with both glucose and insulin intolerance and lipid droplet accumulation. Of note, it was shown that the reduction in STIM1 translocation in this study was due to abnormal O-GlcNAcylation (Arruda et al., 2017). It has been reported that increased O-GlcNAcylation of STIM1 in neonatal cardiomyocytes was linked to a reduction in SOCE (Zhu-Mauldin et al., 2012); however, these studies did not examine the impact of these changes on metabolism and mitochondrial function which should be interrogated.

STIM1 and Orai1 have been shown to regulate whole body metabolism via their regulation of insulin secretion in β-cells. For example, STIM1 and Orai1 knockdown in β-cells leads to reduced glucose-induced insulin secretion (Usui et al., 2019). Of note, STIM1 has been shown to be reduced in expression in islets from type 2 diabetic patients, STZ diabetic mice, and INS-1 cells, resulting in impaired insulin secretion, abnormal Ca^2+^ handling, and ER stress (Kono et al., 2018). Of note, this change may be cell specific, because Orai1 has also been shown to be reduced in lymphocytes from type 2 diabetic patients without changes in STIM1 levels (Wang et al., 2018). These studies suggest that STIM1, Orai1, and SOCE are important for insulin secretion and diabetic cell phenotypes; however, it is unclear whether STIM2, Orai2, and Orai3 contribute to these processes.

In summary, changes in STIM1 and Orai1 isoforms have been shown to alter mitochondrial function and metabolism in various cell types and organs; however, the specific signals connecting them to mitochondrial function and metabolism have yet to be fully elucidated. Furthermore, our knowledge of the potential roles of STIM2 and Orai2/3 in regulating mitochondrial function and metabolism is much less known.

## 6 STIM and Orai in Cell Survival

Cellular Ca^2+^ homeostasis plays a pivotal role in determining cell death and survival. The relationship between Ca^2+^ and cell fate is complex due in part to the fact that Ca^2+^ can act as a stressor and also a second messenger that is involved in multiple pathways in cell death and survival (Orrenius et al., 2003). Consequently, a fine balance between Ca^2+^ depletion and Ca^2+^ overload is key for cell fate determination. A moderate rise of cytosolic Ca^2+^ level promotes cell survival by enhancing mitochondrial bioenergetics and therefore ATP synthesis, as well as activating cell survival signaling, such as Akt and NFAT dependent pathways (Yano et al., 1998; Pu et al., 2003; Rizzuto et al., 2012). Sustained increases in Ca^2+^ level, however, leads to mitochondrial Ca^2+^ overload and subsequent cell death (Naon and Scorrano, 2014). Historically, three processes of cell death were characterized: apoptosis, necrosis, and autophagy. During the past decade or so, new types of cell death such as pyroptosis and ferroptosis have been identified and their importance gradually appreciated (Yu et al., 2021). Indeed, there is growing evidence for a Ca^2+^ related mechanism in ferroptosis in cancerous and noncancerous cells (Chen P. et al., 2020; Angelova et al., 2020), suggesting a unique yet ubiquitous role of Ca^2+^ in general cell death processes. While there is some evidence for members of the less selective TRP cation channels in mediating pyroptosis and ferroptosis (Shi et al., 2021), evidence for STIM/Orai mediated SOCE involvement in these processes are lacking. Therefore, in this section, we will focus on the role of STIM and Orai proteins in the more widely studied cell death pathways, apoptosis, necrosis, and autophagy.

### 6.1 Apoptosis and Necrosis

Apoptosis can be initiated through intrinsic and extrinsic pathways. The intrinsic pathway is activated when there is mitochondrial swelling and/or increased permeability of the mitochondrial membrane, which leads to the release of cytochrome C and cleavage of pro-caspase to caspase 9 (Fesik and Shi, 2001). The extrinsic pathway is activated upon ligand receptor interactions: FasL binding to Fas, or TNFα binding to TNF receptors (Wajant, 2002). STIM/Orai-mediated SOCE has been shown essential in regulating cellular apoptotic pathways with most studies demonstrating its proapoptotic characteristics although there is also evidence for STIM/Orai mediated inhibition of apoptotic signaling (Khadra et al., 2011; Liu et al., 2011; Kondratska et al., 2014).

In a human hepatocarcinoma cell line (HepG2), Yan and colleagues found that ethanol increased intracellular Ca^2+^ level and caused cell damage in a dose-dependent manner (Liu H. et al., 2012), and was associated with increased STIM1 and Orai1 protein levels. In addition, either a SOC inhibitor or a siRNA targeting STIM1 attenuated ethanol induced hepatotoxicity. Subsequent experiments from the same group showed that knockdown of STIM1 and Orai1 significantly restored the mitochondrial membrane potential, decreased cytochrome C release, and attenuated ethanol induced apoptosis (Cui et al., 2015). In a model of hepatic ischemia/reperfusion (I/R) injury, mice lacking STIM1 exhibited an attenuated cellular inflammation and apoptosis compared to controls (Li et al., 2018). In neuronal cells STIM/Orai has also been shown to regulate apoptosis. Rao *et al.* showed that, in hippocampal HT-22 cells, application of hydrogen peroxide (H_2_O_2_) led to significant Ca^2+^ overload and mitochondrial dysfunction, which was attenuated by an SOC inhibitor or a siRNA knockdown of STIM1 (Rao et al., 2013). In a traumatic brain injury model, Hou *et al.* also found that knockdown of STIM1 significantly inhibited apoptotic cell death (Hou et al., 2015), suggesting a role for STIM1 in regulating apoptosis and cell death signaling.

Orai-mediated apoptosis has also been studied in a variety of pathophysiological settings. For example, Flourakis *et al.* identified, that Orai1 was the main source for Ca^2+^ influx in prostate cancer cells (Flourakis et al., 2010). They reported that knockdown of Orai1 protected cells from apoptosis induced by TNFα or Cisplatin whereas Orai1 rescue re-established the normal rate for apoptosis in these cancer cells. It is important to note that although STIM1 expression remained stable when apoptosis was induced, STIM1-Orai1 coupling was required for the pro-apoptotic effects. Given the importance of Orai1 in regulating immune cell function the majority of studies have focused on the role of Orai1 in mediating immune cell apoptosis (Feske, 2009). Using an Apolipoprotein E knockout mouse model, Liang *et al.* demonstrated that silencing Orai1 led to decrease apoptosis in macrophages, which resulted in less foam cell formation and decreased vascular inflammation (Liang et al., 2016). Kim *et al.* showed reduced mitochondrial Ca^2+^ uptake and altered proapoptotic/antiapoptotic gene expression in CD4+ T cells from Orai1-KO mice and provided evidence that NFAT-mediated cell death pathway was the main downstream target for Orai1 mediated Ca^2+^ influx in T cells (Kim et al., 2011). In addition, Orai1 deficient T cells showed increased survival following adoptive transfer to host. Collectively these studies suggest that STIM1/Orai1 mediated SOCE plays an essential role in regulating the intrinsic/mitochondrial pathway for apoptosis.

In contrast, other studies have demonstrated an anti-apoptotic role for STIM1/Orai1, mainly via the extrinsic apoptotic pathway. For example, in Panc1 pancreatic adenocarcinoma cell line knockdown of STIM1 and/or Orai1 increased apoptosis induced by 5-FU or gemcitabine (Kondratska et al., 2014). They also reported that 5-FU and gemcitabine increased SOCE via upregulation of Orai1 and STIM1. Knockdown of Orai1 was also shown to increase apoptosis in glioblastoma cells (Liu et al., 2011).

The apparent contradiction between the pro and anti-apoptotic effects of STIM/Orai-mediated SOCE could be explained in part by the fact that different cancer cell types have varying expression levels of Orai1 and STIM1. In addition, depending on the specific types of stimuli, different intracellular signaling pathways regulated by STIM1/Orai1, may be triggered thereby resulting in different outcomes. In noncancerous cells the anti-apoptotic characteristics of STIM1/Orai1 were also reported. Khadra and colleagues performed a series of experiments showing that, in response to activation of the death receptor CD95, Orai1, and STIM1 colocalize with CD95 and recruit PKCβ2 to the death receptor inducing signaling complex, thus preventing caspase activation and apoptosis (Khadra et al., 2011). In dopaminergic neurons, knockdown of STIM1 led to increased ER stress and apoptosis through PKB inhibition (Selvaraj et al., 2012). In addition to STIM1/Orai1, other STIM/Orai homologues may also play a role in regulating apoptosis. For example, Sobradillo and colleagues investigated Ca^2+^ related mechanisms for colon cancer and found that STIM2 expression was significantly decreased in cancer cells. They also showed that in normal mucosal cells STIM2 knockdown increased resistance to apoptosis (Sobradillo et al., 2014). Tu *et al.* showed in cultured cardiomyocytes that STIM2 expression was significantly increased following I/R injury; whereas knockdown of STIM2 preserved mitochondrial function and attenuated the activation of apoptotic signaling in response to I/R (Tu et al., 2020). There is also evidence for Orai3 mediated regulation of apoptosis, primarily in cancer cells. For example, in breast cancer cells and tissue, Orai3 expression was significantly higher and that knockdown of Orai3 led to cell cycle arrest and apoptosis (Faouzi et al., 2011). Dubois and colleagues introduced a novel channel consisting of Orai1/Orai3 heterodimer and demonstrated its role in prostate cancer cell proliferation (Dubois et al., 2014). They found that prostate cancer cells can undergo an “oncogenic switch.” The increase in Orai3 expression and alterations of tumor microenvironment leads to an increased heteromerization of Orai1 and Orai3, which contributes to the phenotypic transition from SOCE, which is pro-apoptotic, to an Orai1/Orai3 channel that is pro-proliferative. Future studies are needed to decipher the mechanism(s) underlying Orai/STIM mediated regulation of programmed cell death under different conditions.

Unlike apoptosis, necrosis is by in large not a process of programmed of cell death. Although mechanistic studies are lacking, there is evidence that STIM/Orai-mediated SOCE may also be involved in necrotic cell death. Gombedza and colleagues investigated the effect of the internalization of stone-forming calcium crystals on Ca^2+^ signaling in human proximal tubular cells (Gombedza et al., 2019). Amongst other findings, they observed increased cellular necrosis that was accompanied by increased SOCE. They also generated a STIM1 transgenic mouse model in which STIM1 was overexpressed in the skeletal muscle, which increased both SOCE and necrosis in the myofibers of the transgenic mice (Goonasekera et al., 2014). To determine the role of Orai1 in pancreatic acinar cell injury and acute pancreatitis, Wen *et al.* transfected Orai1 into human and mouse acinar cells and found that the application of Orai1 inhibitors prevented acinar cell necrosis (Wen et al., 2015), suggesting a role of Orai1-mediated Ca^2+^ overload in acute pancreatic cell necrosis. Although it is well known that Ca^2+^ overload can lead to not only apoptosis, but also necrosis (Rizzuto et al., 2003; Shaheen et al., 2011), it remains unclear how STIM/Orai-mediated SOCE contributes to necrotic processes. One way in which STIM/Orai may contribute to necrotic processes could be through opening of the mitochondrial permeability transition pore (mPTP). It is well established that necrosis is associated with the opening of the mPTP. It has been shown by He et al., that siRNA-mediated knockdown of STIM1 in H9C2 cardiomyocytes resulted in reduced mPTP opening and reduced ROS (He et al., 2017). However, a definitive role for STIM and Orai members in regulating mPTP opening has yet to be established and would shed additional light on mitochondrial-ER Ca^2+^ regulatory mechanisms.

### 6.2 Autophagy

Autophagy is a tightly regulated physiological process by which cellular components are degraded and recycled, mainly through a lysosome-dependent mechanism (Feng et al., 2014). There are several forms of autophagy, namely macroautophagy, microautophagy, chaperone-mediated autophagy, and crinophagy. As the most well-studied form of autophagy, macroautophagy is a process in which cellular components are covered within a double membrane prior to its fusion with an lysosome, whereas in microautophagy, cellular targets are directly taken up by the lysosome via membrane invagination (Li et al., 2012). Unless otherwise stated, macroautophagy is referred to as autophagy in this section. Studies have shown that Ca^2+^ regulates autophagy through multiple mechanisms (Smaili et al., 2013). In 2007 Hoyer-Hansen *et al.*, demonstrated for the first time that in MCF-7 cells an increase in cytosolic Ca^2+^ induced by various calcium mobilizing agents was a potent activator of autophagy (Hoyer-Hansen et al., 2007). Specifically, they found that thapsigargin, which results in ER Ca^2+^ depletion, the first step in activating STIM1-mediated SOCE, was a potent activator of autophagy via inhibition of mechanistic target of rapamycin (mTOR) in a calcium/calmodulin-dependent protein kinase kinase (CAMKK) dependent manner. On the other hand, Medina *et al.*, concluded that lysosomal calcium was responsible for activation of the Ca^2+^ dependent phosphatase calcineurin, the subsequent dephosphorylation of Transcription factor EB (TFEB) a master transcription factor for autophagy leading to its nuclear localization (Medina et al., 2015). It is worth noting however, that they also showed that thapsigargin was sufficient to induce TFEB nuclear localization, suggesting that ER Ca^2+^ release was sufficient to activate autophagy. These pioneering studies clearly demonstrated a key role for Ca^2+^ in regulating autophagy. While they did not identify the specific Ca^2+^ signaling pathways that were involved, they both showed that ER Ca^2+^ release, an essential step in activating STIM1-mediated SOCE, was sufficient to initiate autophagy.

Zhu *et al.* provided the first direct evidence for SOCE involvement in autophagy, in pancreatic acinar cells (Zhu et al., 2018). In a mouse model of acute pancreatitis induced by Ca^2+^ overload they observed puncta-like colocalization of STIM1 and Orai1 and an increase in SOCE. They observed that the increase in SOCE led to the activation of calcineurin leading to NFAT and TFEB nuclear localization, and subsequent initiation of autophagy. In endothelial progenitor cells, oxidized LDL induced autophagy was accompanied by increased STIM1 leading to activation of CaMKK2 and inhibition of mTOR (Yang et al., 2017). On the other hand, resveratrol a natural polyphenol, activated autophagic cell death in prostate cancer cells, which was associated with reduced STIM1 expression and SOCE (Selvaraj et al., 2016). Inhibition of SOCE had similar effects to resveratrol whereas overexpression of STIM1 reversed the effects. Similarly, knockdown of Orai1 or pharmacological inhibition of SOCE in HepG2 hepatocarcinoma cells potentiated 5-FU induced autophagy whereas overexpression of Orai1 attenuated 5-FU induced autophagic cell death (Tang et al., 2017), suggesting that STIM1 and Orai1 play significant roles in cell death processes including autophagy.

While most of the studies on STIM/Orai related to autophagy have been in the context of cancer, there is also evidence that STIM/Orai is involved in the process of autophagy in normal cells. In neonatal rat cardiomyocytes, the putative STIM1 inhibitor ML9 induced cell death by inducing lysosomal dysfunction and disrupting autophagic flux (Shaikh et al., 2018). However, interpretation of these findings needs to be considered carefully since it is well known that ML9 inhibits several different protein kinases, including myosin light-chain kinase (MLCK), PKA, and protein kinase C (PKC) (Hidaka and Kobayashi, 1992; Takahashi et al., 1997; Smyth et al., 2009). Angiotensin II (Ang II) had been shown to induce cardiomyocyte hypertrophy in an SOCE-dependent manner (Hunton et al., 2002), and more recently it was reported to induce autophagy in neonatal cardiomyocytes in an SOCE and Orai1-dependent manner (Zheng et al., 2021). In the same study, *in vivo* Ang II infusion was shown to increase autophagic flux in the heart and this was attenuated by decreasing Orai1 levels following treatment with an AAV-Orai1-siRNA1.

It is now readily accepted that Ca^2+^ plays a role in regulating autophagy (Kondratskyi et al., 2018) and while some have suggested that TRP channel family of Ca^2+^ channels contribute the regulation of autophagy (Sukumaran et al., 2015; Sukumaran et al., 2016) there is growing support that it is mediated via a STIM/Orai-dependent SOCE pathway. Studies examining the extent to which both STIM1 and Orai1 contribute to the regulation of autophagy are warranted. Nevertheless, it is clearly context dependent since SOCE appears to both activate and attenuate autophagy depending on cell type and the specific stimulus.

## 7 Redox Regulation of STIM and Orai Proteins

Several studies have suggested that STIM and Orai proteins are sensitive to and are regulated by changes in redox status and these changes will be discussed in this section. S-nitrosylation is a significant regulator of redox signaling, which is mediated through increases in nitric oxide (NO) and subsequent covalent attachment of NO to cysteine (Cys) thiols on proteins. Interestingly, it has been shown that neuronal nitric oxide synthase (nNOS), which generates NO, is expressed in the SR (Xu et al., 1999), where STIM1 also resides. It was recently shown by Gui *et al.*, (Gui et al., 2018), that STIM1 undergoes S-nitrosylation on Cys49 and Cys56 and that S-nitrosylation of STIM1 inhibits its oligomerization and reduces SOCE. The same study also showed that genetic and pharmacological reductions in nNOS reduce S-nitrosylation of STIM1 and reversed changes in SOCE. The authors speculated that STIM1 activity and SOCE increase during heart failure because of a reduction in NO bioavailability; however, this has yet to be determined. In addition to S-nitrosylation, STIM1 has also been shown to undergo S-glutathionylation, where it has been shown to have an opposite effect on STIM1 activity and SOCE compared to S-nitrosylation. Hawkins *et al.*, have shown that STIM1 can be S-glutathionylated on Cys56, which increases both the activity of STIM1 and also increases SOCE (Hawkins et al., 2010). In the same study, it was shown that S-glutathionylation of STIM1 modulated mitochondrial bioenergetics and Ca^2+^ handling (Hawkins et al., 2010). One would hypothesize that changes in the balance between s-nitrosylation and S-glutathionylation of STIM1 could perhaps contribute to cardiovascular pathologies although this remains to be determined.

It has also been shown that STIM2 is subject to oxidative modification. For example, Gibhardt *et al.*, (Gibhardt et al., 2020), showed that STIM2 has an additional ten cytosolic cysteine residues in comparison to STIM1. They also showed that upon the induction of oxidative stress the oxidation of Cys313 on STIM2 is modified promoting a reduction in SOCE through the prevention of STIM2 oligomerization. It has also been shown that in response to the NO donor, nitrosoglutathione, STIM2 was S-nitrosylated at cysteines 15, 53, and 60 in HEK cells which was required for the stabilization of STIM2 (Novello et al., 2020). Like STIM isoforms, Orai isoforms are also subject to redox regulation. Of interest, it has been postulated and shown in HEK cells that Orai1 is redox-sensitive and that Orai3 is redox-insensitive (Alansary et al., 2015) this is largely due to the lack of the redox sensor, Cys195 in Orai3. Mutations in the Orai1 redox sensor, Cys195, have been shown to inhibit SOCE in response to the knockdown of the sodium/calcium exchange in HEK293 cells (Ben-Kasus Nissim et al., 2017). Alansary *et al.* have shown that treatment of HEK293 cells with H_2_O_2_ reduces Orai1-mediated SOCE which was shown to be the result of oxidation of Cys195 on Orai1 (Alansary et al., 2016). It has also been shown in HEK293 cells that hydrogen sulfide (H_2_S) treatment inhibits Orai3-mediated SOCE but not Orai1 and Orai2-mediated SOCE. The authors determined that this difference was due to the presence of Cys226 and Cys232 in Orai3 both of which were absent in other Orai isoforms and were shown to mediate the reduction in SOCE in response to hydrogen sulfide (H_2_S) (Fresquez and White, 2021). It is possible that STIM1 coupling with either Orai1 or Orai3 could well depend on cellular redox status and perhaps act as a redox sensor although this remains to be determined. At present, it remains unclear as to the extent to which Orai2 is regulated by redox and oxidative stress; therefore, future studies should be aimed at determining redox modulation of Orai2 and impact on resultant SOCE. In addition, it remains unclear based on these studies how these redox modifications of STIM and Orai isoforms and splice variants impact the function of different cell types and organ systems, which needs to be established moving forward.

## 8 Physiological and Pathophysiological Roles of STIM/Orai in the Cardiovascular System

It is increasingly clear that STIM-Orai signaling is an important regulator of cardiovascular physiology and homeostasis as well as playing a significant role in cardiovascular disease processes, such as hypertrophy, ischemia/reperfusion (I/R), and heart failure. In this section, we will discuss the current knowledge concerning the canonical and non-canonical functions of STIM and Orai proteins in the heart during instances of cardiac pathology and during normal physiology.

### 8.1 STIM1

Until the early 2000s, it was believed that cardiac TRP channels played a key role in the underlying Ca^2+^ signaling associated with cardiac hypertrophy and heart failure (Nakayama et al., 2006). However, upon the discovery of STIM1 and Orai1 as key regulators of SOCE and with STIM1/Orai1-mediated SOCE being associated with cardiac hypertrophy (Ohba et al., 2009; Voelkers et al., 2010; Hulot et al., 2011; Luo et al., 2012), there was a growing appreciation of this signaling mechanism in the heart. In these studies, STIM1, Orai1, and resultant SOCE were all shown to be increased and participate in the activation of calcineurin and the nuclear translocation of NFAT to activate hypertrophic signaling (Ohba et al., 2009; Voelkers et al., 2010; Hulot et al., 2011; Luo et al., 2012). Of note, the study by Luo *et al.*, (Luo et al., 2012), showed that STIM1L, which was very low in healthy adult cardiomyocytes, was significantly increased in response to the hypertrophic agonist, phenylephrine (PE). The authors speculated that this increase in STIM1L was a stimulus for the induction of the fetal gene program. Despite this, little remains known regarding the function of STIM1L in the heart. Since the establishment of a role for STIM1/Orai1 in cardiac hypertrophy, several additional studies have tried to examine the mechanisms driving the increase in STIM1 during hypertrophy. One such study in neonatal cardiomyocytes treated with PE showed that a decrease in expression of miR-223 was responsible for an increase in STIM1 expression and subsequent increased hypertrophic signaling, which was shown to also involve changes in GSK3β, β-catenin, and SRY-box transcription factor 2 (SOX2) (Zhao et al., 2018). Increased phosphorylation of STIM1 by Fam20c Golgi associated secretory pathway kinase (Fam20c) was also shown to contribute to increased STIM1 expression and increased SOCE during pressure-overload (Pollak et al., 2018), suggesting that several signaling mechanisms are contributing to the increase in STIM1 during pressure overload.

Rare, heterozygous gain of function mutations in STIM1 in humans result in complex neuromuscular phenotypes, including some cardiac involvement (Walter et al., 2015; Harris et al., 2017). Studies of patients undergoing cardiac catheterization found SNPs in the STIM1 gene, which correlated with metabolic defects, ER stress, and an increase in mortality (Kraus et al., 2015). More recently, mutations have been shown that impact STIM1 expression. For example, it has been shown that a variant upstream of STIM1, named rs3061890, has been associated with coronary artery disease and has been shown to repress STIM1 in an ELF1-dependent manner (Zhang et al., 2021). Collectively, these studies suggest that STIM1 has an important role in mediating cardiovascular disease although the regulatory mechanisms governing STIM1 expression and activity in the heart need to be further established.

After the establishment of its role in cardiac hypertrophy, there was significant controversy in the field as to the relevance of STIM1/Orai1-mediated SOCE in the non-diseased heart due to the predominant regulation of cardiac Ca^2+^ handling on a beat-by-beat basis by voltage-gated Ca^2+^ handling; therefore, the field began to focus their attention on determining the physiological role of both STIM1 and Orai1 in the heart. We performed the initial phenotyping of the constitutive cardiomyocyte-specific STIM1-KO mouse in 2014 (Collins et al., 2014). We showed that cardiomyocyte STIM1 was an essential regulator of ER/SR and mitochondrial function as STIM1-KO mice had a progressive dilated cardiomyopathy associated with significant ER stress, the presence of abnormally shaped and distributed mitochondria, and changes indicative of increased mitochondrial fission. Using the same mice, we later showed that cardiomyocyte STIM1 was also an important regulator of cardiac metabolism as KO mice had significant perturbations in both cardiac glucose and fatty acid oxidation (Collins et al., 2019).

STIM1 has been further linked to regulating ER stress and mitochondrial-mediated apoptosis in the heart. Using Cardiomyocyte-specific STIM1-KO and overexpression mice treated with doxorubicin, Zhu *et al.*, have shown that mice lacking STIM1 have increased cardiac injury in response to doxorubicin treatment which was associated with increased apoptosis and increased GRP78-mediated ER stress, and was essentially reversed in the STIM1 overexpression model (Zhu et al., 2021). Interestingly, in the same study, doxorubicin treatment of wild-type mice suppressed STIM1 expression and SOCE, suggesting that STIM1 and SOCE could contribute to doxorubicin-mediated cardiotoxicity although this currently remains undetermined. Several additional transgenic mouse studies have been performed to examine the function of cardiomyocyte STIM1. One of these studies by Parks *et al.*, (Parks et al., 2016), examined the impact of inducible cardiomyocyte-specific STIM1-KO in mice and showed a phenotype very similar to the constitutive cardiomyocyte-specific STIM1-KO mice (Collins et al., 2014). However, unlike earlier studies, this study showed that KO mice had a blunted hypertrophic response to pressure overload. Ohba *et al.* (Ohba et al., 2017) examined the impact of heterozygous STIM1-KO and found that these mice were essentially normal at baseline, but increased mortality was observed in the KO in response to 4-weeks TAC despite lack of hypertrophy and induction of fetal gene expression. The authors concluded that a partial lack of STIM1 resulted in abnormal responses to cardiac stress; however, this model was a whole-body STIM1 heterozygous model rather than cardiomyocyte specific. The Houser lab also showed that STIM1-mediated Ca^2+^ influx during hypertrophy contributed to action potential prolongation, SR Ca^2+^ overload, Ca^2+^ sparks, and CaMKII-mediated cell death determined using a combination of pharmacological inhibition of STIM1 with BTP2 or a dominant-negative Orai1 construct in cardiomyocytes from banded felines (Troupes et al., 2017). These studies clearly suggest an important role of STIM1 in cardiovascular disease processes.

In addition, changes in STIM1, STIM1L, and Orai1 have been observed in a pulmonary hypertension model of monocrotaline-induced RV-hypertrophy in which STIM1 expression was reduced but the expression of both STIM1L and Orai1 were increased and this was associated with significant changes in Ca^2+^ handling (Sabourin et al., 2018). Examination of cardiac overexpression of STIM1 has also shed light on the physiological role of STIM1. Studies by Molkentin and colleagues (Correll et al., 2015) showed that cardiomyocyte-specific overexpression of STIM1 not only resulted in increased Ca^2+^ entry but also promoted cardiac hypertrophy, decreased cardiac function, increased mortality, and increased fetal gene expression which was associated with mitochondrial ultrastructural abnormalities and significant alterations in Ca^2+^ handling (i.e., spontaneous Ca^2+^ transients, increased Ca^2+^ spark frequency, increased diastolic Ca^2+^, and remodeling of the L-type Ca^2+^ channel (LTCC) current). Like the studies in KO mice, the responses to hypertrophic stimuli in these mice were significantly exacerbated. Given that it appears too much or too little cardiomyocyte STIM1 results in similar cardiovascular phenotypes, it is likely that STIM1 levels are tightly regulated and play significant roles in the precipitation of cardiovascular diseases.

STIM1 has also been implicated in contributing to cardiomyocyte injury. For example, reductions in STIM1 levels in hypoxia/reoxygenated cardiomyocytes have been shown to mediate reduced mitochondrial Ca^2+^ overload, in addition to reduced mPTP opening and reduced ROS generation (He et al., 2017). The protective effects of resveratrol following hypoxia/reoxygenation experiments in isolated neonatal rat cardiomyocytes (NRCMs), which included reduced apoptosis and reduced Ca^2+^ overload were also attributed to inhibition of STIM1 (Xu et al., 2019); however, given that resveratrol has multiple cellular affects its direct link to STIM1 inhibition should be considered with caution. Although it should be noted that increasing STIM1 levels was shown to exacerbate the injury in the same model (Xu et al., 2019).

STIM1 has also been shown to have additional roles in the heart other than its roles in hypertrophic signaling and regulation of ER/SR-mitochondrial function. One of these roles is in regulating cardiac electrophysiology. For example, the Rosenberg group showed that STIM1 is expressed in the sinoatrial node (SAN) where it regulates SAN function through modulation of SOCE and LTCC and the regulation of heart rate and cholinergic responsiveness (Zhang et al., 2015a). The same group using STIM1 reporter mice subsequently showed that STIM1 regulates conduction from the SAN to the coronary sinus (Zhang et al., 2020). They showed that STIM1 was an important regulator of atrial function, interatrial conduction, and arrhythmic activity as mice lacking STIM1 in coronary sinus cardiomyocytes showed reductions in conduction and increased arrhythmogenesis. In addition, Bonilla *et al.*, (Bonilla et al., 2019), have shown that spontaneous Ca^2+^ sparks in the setting of cholinergic stress were reduced through STIM1 inhibition with SKF-96365 and in STIM1-KO cardiomyocytes. These observations were similar to those seen in hearts of mice with catecholaminergic polymorphic ventricular tachycardia. We have also shown that hearts from cardiomyocyte-specific STIM1-KO mice have altered heart rates and significant QT prolongation which could be the result of changes in the downstream targets of the cardiomyocyte kinome, leading to potential crosstalk with existing ionic channels that regulate the cardiac action potential such as the LTCC (Collins et al., 2022). Recently, it was hypothesized that the increased and early mortality reported in cardiomyocyte-specific STIM1-KO mouse models could be due to increased arrhythmogenic activity. In support of this, it was shown that hearts from inducible cardiomyocyte specific STIM1 knockdown mice had increased arrhythmic activity and discordant action potential alternans (Cacheux et al., 2019). It is likely that these arrhythmias are due to STIM1 regulating existing action potential currents such as the LTCC although this needs to be determined. Clearly, further studies are required to fully determine the role of STIM1 in regulating cardiac electrophysiology and how STIM1 interacts with other ionic channels known to regulate the electrical activity of the heart and to determine additional non-conical roles of STIM1.

### 8.2 Orai1

Studies in Zebrafish were amongst the first to indicate a significant role of Orai1 in the heart, where Orai1 deletion resulted in the development of heart failure and significant ultrastructural defects (Volkers et al., 2012). Despite this, conflicting results have been observed in mouse models. Cardiomyocyte-specific KO of Orai1 did not give rise to a significant phenotype at baseline as contractile function and cardiac hemodynamics were both normal; however, when subjected to the hypertrophic agonist, Ang II, cardiomyocyte size and fibrosis were increased contributing to exacerbated Ang II-dependent cardiac remodeling (Segin et al., 2020). Interestingly, this exacerbation was associated with reductions in both STIM1 and Orai3 expression. Interestingly, conflicting results were observed by Bartoli *et al.* who used cardiomyocyte specific Orai1 mutant mice with a mutation in the Orai1 pore and found that inhibition of Orai1 activity during TAC resulted in preserved cardiac function and preserved Ca^2+^ handling (Bartoli et al., 2020). It has also been shown that Orai1 may contribute to hypertrophy associated with diabetic cardiomyopathy through the regulation of Drp-1-dependent mitochondrial fission (Wu et al., 2021). Specifically, inhibition of Orai1 was shown to reduce cardiac hypertrophy and improve mitochondrial function through reductions in Drp-1, calcineurin, and ERK1/2 activities. Like STIM1, these studies suggest that Orai1 has a significant role in maintaining cardiac homeostasis; however, in the same respect as STIM1, additional studies are required to determine to fully appreciate the physiological role of Orai1 in the heart.

### 8.3 Other STIM/Orai family members

For several years, the focus has been on the roles of both STIM1 and Orai1 in the physiological and pathological regulation of the heart, with little to no focus on other members of the STIM/Orai families. However, in recent years, STIM2, Orai2, and Orai3 isoforms have also been shown to be present in the heart and appear to have important regulatory roles. Of these additional isoforms, Orai3 has been the most interrogated in the heart. Saliba *et al.* showed that Orai3 activity was increased in hypertrophic cardiomyocytes (Saliba et al., 2015). Specifically, the authors showed that Orai3 was the preferred partner of STIM1 over Orai1 during established hypertrophy and that increased Orai3 activity was responsible for an increase in an arachidonic acid activated Ca^2+^ channel activity during hypertrophy. In a later study by the same group, they showed that the store-independent channel activity mediated by Orai3 during hypertrophy was largely driven by inflammation mediated by TNFα and CD11b/c cells (Keck et al., 2019). More recently, the physiological role of Orai3 has been interrogated in the heart using a cardiomyocyte-specific deletion. Gammons *et al.* (Gammons et al., 2021) showed that both constitutive and inducible cardiomyocyte-specific deletion of Orai3 develop a phenotype consistent with dilated cardiomyopathy, with increased fibrosis, increased mortality, ultrastructural changes in mitochondria, increased mitochondrial fission, and abnormal sarcomeric structure; highlighting a potentially important regulatory role of Orai3 in the heart.

A recent study examining the expression of STIM and Orai isoforms in human failure samples indicated that in addition to the expression of STIM1 and Orai1 being increased and decreased in the left ventricles of heart failure patients, respectively, the expression of STIM2, Orai2, and Orai3 remained unchanged (Cendula et al., 2019). The reduction in Orai1 levels was restricted to male patients, suggesting that sex differences could exist in the expression of STIM-Orai family members, which have not been examined in detail and could contribute to documented sex differences that exist in heart failure progression. Interestingly, in this same study, the STIM2 splice variant STIM2.1, shown previously to have an inhibitory effect on SOCE in T-cells (Miederer et al., 2015), was shown to be significantly reduced in LV of HF patients with a reduction in the ratio of STIM2.1/STIM2. The authors proposed this was indicative of a switch to the stimulatory form of STIM2, STIM2.2. In a cell culture model of cardiomyocyte I/R injury it has been shown that STIM2 expression is upregulated without change in STIM1 expression, and STIM2 knockdown was associated with reduced levels of apoptosis, reduced mitochondrial Ca^2+^ overload, and preserved mitochondrial function (Tu et al., 2020). In skeletal muscle STIM2 colocalizes and interacted with calsequestrin to modulate diastolic Ca^2+^ and Ca^2+^ buffering; however, it remains to be determined whether this also occurs in cardiomyocytes (Jeong et al., 2021). It is possible that targeting STIM2 isoforms could be an important therapeutic strategy in models of cardiomyocyte injury.

While there is growing evidence for a role of STIM2 and Orai3 in the heart future studies are required to establish their physiological roles in the heart and how they are involved in regulating cardiac hypertrophy and I/R injury. Moreover, our understanding of the importance of STIM2 splice variants is in its infancy. In addition, even though Orai2 is present in cardiomyocytes there have yet to be any studies examining the physiological or pathophysiological role of Orai2 in the heart. Clearly future studies are required to better elucidate the cardiovascular functions of all STIM/Orai isoforms and splice variants and their roles in mediating cardiovascular disease.

### 8.4 Roles of STIM and Orai proteins in non-cardiomyocyte cells in the heart

In addition to cardiomyocytes, STIM and Orai proteins are also widely expressed in other cell types present in the heart, including endothelial cells, vascular smooth muscle cells (VSMCs), and fibroblasts, where they have different effects on the functionality of each cell type. For example, it has been shown that Orai1-mediated SOCE is important for endothelial cell function since endothelial cells with Orai1 knockdown or inhibition have been shown to have reduced tube formation and migration (Li et al., 2011). In this study Orai1 disruption also reduced VEGF-mediated Ca^2+^ entry. Interestingly, STIM/Orai signaling is increased in conditions of high glucose (i.e., 25 mM) in endothelial cells where STIM1, STIM2, Orai1, Orai2, and Orai3 are all increased in addition to an increase in SOCE through calcineurin/NFAT-dependent mechanisms (Daskoulidou et al., 2015). On the other hand, coronary endothelial cells from diabetic mice had lower STIM1 levels, which was linked to impaired endothelial relaxation; this was reversed by partially restoring with adenoviral STIM1 vector (Estrada et al., 2012). These studies suggest that STIM and Orai proteins are important for endothelial cell function in the heart; however, more studies are required to examine the precise functions of STIM/Orai proteins and their respective splice variants in these cells.

STIM1/Orai1-mediated SOCE has been implicated in regulating VSMC function in the heart. Studies by Guo *et al.*, (Guo et al., 2012), have shown that siRNA silencing of both STIM1 and Orai1 not only reduced SOCE but also prevented Ang II-mediated cell proliferation and reduced Ang II-mediated neointimal growth in response to balloon injury. This reduction could be the result of changes in the expression of Orai1 interacting partners, such as SOCE-associated regulatory factor (SARAF) and Homer. It was recently shown that the expression of SARAF was increased in balloon injured arteries along with STIM1 and Orai1. SARAF was shown to specifically regulate Orai1-mediated VMSC proliferation in response to balloon injury (Martin-Bornez et al., 2022). Homer has also been shown to colocalize with STIM1/Orai1 and regulate Orai1-mediated VSMC proliferation and neointimal growth in the setting of balloon injured arteries (Jia et al., 2017). Furthermore, VSMC remodeling in response to hypertension is associated with a decrease in L-type Ca^2+^ channels (LTCC) and a reciprocal upregulation of STIM1 and Orai1 (Johnson et al., 2020). In addition, inhibition of LTCC was found to activate STIM1/Orai1 Ca^2+^ entry potentially contributing to a proliferative phenotype; however, the mechanism by which this occurs is currently unknown (Johnson et al., 2020). Interestingly, smooth muscle cell-specific KO of STIM1 resulted in smaller myocardial infarct size following coronary artery occlusion and reperfusion (Mali et al., 2018), which was associated with reductions in ER stress, reductions in both p38 and ERK1/2 signaling, reduced apoptosis, reduced fibrosis, and reduced inflammation (Mali et al., 2018). However, the mechanisms by which smooth muscle cell STIM1 contributes to these processes following myocardial I/R remain to be determined. Mice with smooth muscle cell-specific KO of STIM1 have also been shown to have reduced myogenic tone with higher plasma levels of catecholamines and significant dysregulation of the cytoskeleton (Pichavaram et al., 2018). Recently, it was shown that mice with inducible VMSC-specific STIM1-KO had reductions in colocalization of Ca^2+^ clusters between the SR and PM. In addition, these mice were shown to be hypotensive with reduced contractility in resistance arteries (Krishnan et al., 2022). Collectively, these studies highlight the importance of STIM/Orai signaling in VMSCs which should be expanded upon in future studies including determining the roles of other isoforms (i.e., STIM2, Orai2, and Orai3) and their splice variants.

Evidence suggests that STIM/Orai-mediated SOCE may regulate cardiac fibroblast activity during induction of cardiac hypertrophy and the development of heart failure. Increased SOCE has been observed in fibroblasts from failing hearts which was associated with an increase in Orai1 expression, increased colocalization with STIM1, and associated with increased fibrosis (Ross et al., 2017). In addition, Zhang *et al.*, (Zhang et al., 2016), have shown that upregulation of fibronectin, connective tissue growth factor, smooth muscle α-actin, and smad2/3-dependent signaling seen in response to Ang II treatment could be blocked independently using both the SOCE inhibitor, SKF-96365, and STIM1/Orai1 knockdown in cardiac fibroblasts. Collagen synthesis and fibroblast proliferation have both been shown to be reduced in the setting of reduced Orai-mediated SOCE in human cardiac fibroblasts (Chen et al., 2021). This study suggests that STIM1/Orai1 may regulate cardiac fibroblast activity and activation. It has been shown that SOCE may increase in aged human cardiac fibroblasts which was associated with a reduction in the expression of pro-fibrotic sprouty homologue 1 (Spry1) possibly contributing to senescence-mediated fibrosis in the heart; however, in these studies expression levels of STIM/Orai were not changed (Mohis et al., 2018) so the catalyst driving the increase in SOCE in this study remains unclear. Moving forward, it will be important better understand the different functions and roles of STIM and Orai isoforms in the various cell types of the heart and whether these STIM/Orai isoforms contribute to crosstalk between these cell types during physiological and pathophysiological instances.

## 9 STIM/Orai in Neurodegeneration

In the adult mouse brain, STIM1 exhibits the highest expression in cerebellum and relatively lower expression in the cerebral cortex, whereas STIM2 is predominantly expressed in the cortex (Moccia et al., 2015). In addition, all three Orai isoforms can be detected in the mouse brain (Moccia et al., 2015). Orai1 appears to be expressed at low levels across multiple brain regions. Orai2, on the other hand, exhibits increased expression levels in the cerebellum as well as the hippocampus. Orai3 is strongly expressed in the cerebellum (Lein et al., 2007). In 2009, Venkiteswaran and Hasan demonstrated that STIM1 and Orai1 were necessary for normal flight and rhythmic firing of the flight motoneurons in *Drosophila* (Venkiteswaran and Hasan, 2009). Following this study there was growing evidence that STIM/Orai plays an important role in neuronal physiology. Interestingly, there are a couple of studies showing that STIM1 regulates Cav1.2, a voltage-gated calcium channel ubiquitously expressed in neurons, cardiac muscles, and smooth muscle cells (Park et al., 2010; Wang et al., 2010). Neuron-specific roles for STIM/Orai include regulating axonal growth, maintaining synaptic plasticity, as well as modulation of memory formation. The role of SOCE in neuronal function has been extensively reviewed elsewhere (Kraft, 2015; Moccia et al., 2015). Here, we review current understandings of STIM/Orai proteins in neurodegenerative processes with a specific highlight on transgenic models.

### 9.1 Trauma induced neurodegeneration

Two phases of damage can be caused by trauma to the brain. Primary damage occurs at the time of injury whereas secondary damage can last hours to months following the initial impact (Algattas and Huang, 2013). Our current understanding of the mechanism(s) underlying secondary traumatic brain damage emphasizes increased Ca^2+^ influx induced by substantial release of excitatory neurotransmitters, primarily glutamate (Weber et al., 1999). Previous studies have demonstrated the significance of both voltage-gated calcium channels and store-operated calcium entry in mediating trauma-induced cell damage in the brain (Weber, 2012). In an *in vivo* diffuse axonal injury model, neuronal STIM1 protein levels showed a time-dependent increase peaking at 12 h after injury (Li Y. et al., 2013). The authors speculated that this increase in STIM1 might contribute to neuronal necrosis via an increase in SOCE. This concept was supported in an *in vitro* traumatic neuronal injury model by Hou *et al.* who showed that following traumatic injury induced by stainless steel punch cut, STIM1 expression in mouse cortical neurons significantly increased and peaked between 6 and 12 h (Hou et al., 2015). The role of STIM1 in neuronal injury in this model was confirmed by the observation that knockdown of STIM1 decreased neuronal apoptosis, increased viability, and attenuated glutamate receptor 1 (GluR1)-mediated increase of Ca^2+^ in the cytoplasm (Hou et al., 2015). On the other hand, Rao *et al.* showed that in an *in vivo* cortex injury model STIM2 expression was increased up to 12 h following injury whereas, STIM1 expression remained unchanged (Rao et al., 2015). Moreover, they showed that knockdown of STIM2, but not STIM1, provided protective roles in neuronal survival following injury (Rao et al., 2015). These findings are similar to reports that STIM2 rather than STIM1 contributes to ischemia-induced neuronal injury (Berna-Erro et al., 2009). The differences between these studies may be in part due to different *in vitro* and *in vivo* models for inducing neuronal injury. However, different cell distribution as well as potentially different functions of STIM1 and STIM2 may also be contributing factors. For example, STIM2 has been implicated in regulating basal neuronal Ca^2+^ levels, whereas it has been suggested that STIM1 is an important regulator of mGluR1-mediated Ca^2+^ signaling (Zhang and Hu, 2020). Similar to the cardiomyocyte studies, targeting the balance between STIM1 and STIM2 levels should be explored in more detail. Clearly, more studies are warranted to further understand how STIM1 and STIM2 are involved in trauma induced brain damage.

### 9.2 Ischemia induced neurodegeneration

Cerebral ischemia can be induced by a variety of events that cause inadequate oxygen delivery to the brain, including thrombotic stroke, embolic stroke, or systemic hypoperfusion. Low oxygen delivery to the brain leads to an increase in glutamate release and subsequent stimulation of glutamate receptors, primarily NMDARs, with the resulting Ca^2+^ overload leading to neuronal death. In a transient cerebral ischemia model where the middle cerebral artery was occluded for 1 h, infarct volume in mice lacking STIM2 was significantly lower than that of control mice 24 h later (Berna-Erro et al., 2009). Consistent with the *in vivo* data, they also reported that *in vitro* culture of brain tissues from STIM2 knockout mice exhibited increased survival following hypoxia. These protective effects were not observed in primary neuron cultures from either STIM1-deficient mice or Orai1-deficient mice. Interestingly, however, in wildtype mice that were transplanted with STIM1-deficient bone marrow, there was a 70% reduction in infarct size following the same 1-h middle cerebral artery occlusion, suggesting that the protective roles for STIM1 in ischemia-induced brain damage are likely due to its function in hematopoietic cells rather than neurons (Braun et al., 2009). Similar protective results were observed in Orai1-deficient bone marrow chimeras (Varga-Szabo et al., 2008).

Zhang *et al.* showed in a rat model of global cerebral ischemia model that STIM1 and Orai1 expression significantly increased following injury and peaked on day 4. Additionally, STIM1 siRNA injection significantly improved neurological functions and decreased neuronal Ca^2+^ levels following ischemia, suggesting a role for STIM1 in mediating ischemia-induced brain damage (Zhang M. et al., 2014). Orai2-deficient mice also exhibited diminished Ca^2+^ signals following oxygen deprivation and were protected from neuronal damage resulting from transient middle cerebral artery occlusion (Stegner et al., 2019). Some of the injury that occurs with this model involves T-cell mediated inflammation and Orai2 is known to regulate Ca^2+^ influx in T-cells. However, bone marrow transplant studies showed that the protective effects of a lack of Orai2 was independent of T cells (Stegner et al., 2019), suggesting a protective role for Orai2 in this setting.

While most reports link increased STIM/Orai levels to neuronal injury, Secondo *et al.*, reported that STIM1 and Orai1 expression levels decreased both in an *in vitro* hypoxia/reoxygenation model and an *in vivo* and focal ischemia model of stroke (Secondo et al., 2019). In addition, an ischemic preconditioning protocol prevented STIM1 and Orai1 downregulation. Moreover, siRNA knock down of either STIM1 or Orai1 attenuated the protection associated with ischemia preconditioning. It was proposed that the neuroprotection resulting from increased STIM1 and Orai1 was due to maintaining ER Ca^2+^ homeostasis thereby reducing ER stress (Secondo et al., 2019). There is increasing evidence of a role for STIM/Orai protein in ischemic injury; however, as with traumatic neuronal injury there are discordant results regarding the role of specific isoforms. The use of global KO models has a potentially confounding effects, given the different cell types in the brain. The development of inducible cell type specific STIM/Orai transgenic and KO models will be needed to improve our understanding of their specific roles in ischemic brain injury.

### 9.3 Alzheimer’s Disease

Alzheimer’s disease is a progressively worsening neurodegenerative disease that accounts for over 60% cases of dementia. While most cases of Alzheimer’s disease are sporadic and have a relatively late onset, around 1–2% of cases are a result of an autosomal dominant genetic disease. In these familial cases onset of disease is significantly earlier (early 50s vs. over 65) (Lane et al., 2018). Although the pathological processes between sporadic forms and familial Alzheimer’s disease are similar, different disease associations do exist. For instance, ApoE2 allele is associated with decreased risk of sporadic Alzheimer’s disease whereas individuals with ApoE4 allele have increased disease risk. The familial form of Alzheimer’s disease is associated with several proteins, including amyloid beta precursor protein, presenilin 1 and presenilin 2 (Lane et al., 2018).

Over the past two decades or so, numerous studies have demonstrated the involvement of Ca^2+^ homeostasis and intracellular signaling in the development of Alzheimer’s disease (Woods and Padmanabhan, 2012; Tong et al., 2018). By comparing intracellular Ca^2+^ levels in hippocampal neurons isolated from young and mid-aged mice, Raza *et al.* provided evidence that aging neurons have significantly higher basal levels of intracellular Ca^2+^, suggesting that altered Ca^2+^ homeostasis may be a mediator for aging related neuronal deficits (Raza et al., 2007). Ca^2+^ dysregulation has been associated with cascading events of Alzheimer’s disease with evidence that it can precede detectable pathological changes (Chakroborty et al., 2009; Muller et al., 2011). Specifically, inositol triphosphate and ryanodine receptor mediated Ca^2+^ signaling was shown to play pivotal roles in AD of transgenic mouse models as well as human cells (Stutzmann et al., 2007; Cheung et al., 2010). There is growing evidence for decreased SOCE and downregulated STIM/Orai proteins in both the sporadic form and the familial form of Alzheimer’s disease. For example, there was up to 70% decrease in STIM1 levels in brain tissues from sporadic Alzheimer’s disease patients compared to control (Pascual-Caro et al., 2018). STIM1 knockout in a neuroblastoma cell line showed that although STIM1 was not required for neuronal cell differentiation, it was required for cell survival (Pascual-Caro et al., 2018). In the familial form of Alzheimer’s disease, SOCE was found to be attenuated and endogenous presenilin 1 interacted with STIM1 in the ER (Tong et al., 2016). These results suggest that STIM1 may be a therapeutic target for Alzheimer’s disease which should be interrogated further. Of note, a recent study from Niemeyer and colleagues identified a splice variant of STIM1, STIM1B, and showed it was significantly decreased in both familial and sporadic forms of AD (Ramesh et al., 2021). Although possessing higher expression level in the cortex and hippocampus, the role of STIM2 in the disease progression of Alzheimer’s disease has been less studied. A body of work from Bezprozvanny and colleagues, however, provided insights to the role of STIM2 in disease models for Alzheimer’s (Zhang et al., 2015b; Popugaeva et al., 2015). They showed both *in vitro* and *in vivo* that overexpression of STIM2 attenuated mushroom spine loss induced by Ab42 oligomers and provided evidence for STIM2-mediated maintaining of calmodulin kinase II activity. These studies provided support for targeting STIM proteins in Alzheimer’s disease; although, several key questions remain such as the role of Orai proteins in Alzheimer’s disease. There is evidence that overexpression of both STIM2 and Orai1 increased neuronal Ca^2+^ level; however, no sign of neurodegeneration was observed, suggesting a dissociation of SOCE machinery with Alzheimer’s disease progression as determined by amyloidogenesis and immunohistochemistry (Majewski et al., 2020). Of note, another study showed, in a cell model of Alzheimer’s disease, that downregulation of Orai2 increases SOCE and decreases amyloid-beta accumulation, which suggests a potential benefit for Orai2 knockdown in Alzheimer’s disease (Scremin et al., 2020). In addition, although the majority of current evidence suggest relationships between STIM proteins and Alzheimer’s disease progression, more work needs to be done to decipher the roles of STIM/Orai-mediated calcium entry as well as STIM/Orai independent-SOCE in Alzheimer’s disease.

### 9.4 Huntington’s and Parkinson’s Disease

Huntington’s disease is a progressive neurodegenerative disorder that typically presents as chorea, depression, and dementia. It is caused by an expansion of CAG trinucleotide repeats in the resulting in mutant huntingtin (mHTT) gene. It is well acknowledged that overstimulation of glutamate receptors as well as the downstream effects on Ca^2+^ signaling plays a major role in neuronal death in Huntington’s disease (Mccolgan and Tabrizi, 2018). Studies have shown that SOCE is involved in the pathogenesis of Huntington’s disease and that abnormal SOCE leads to dysregulated synaptic response (Wu et al., 2011; Wu et al., 2016; Wu et al., 2018). Of note, STIM2 appears to play an important role in the disease process. Wu *et al.* showed increased activity of IP_3_/IP_3_R1 pathway and overexpression of STIM2 in neurons of a mouse model of Huntington’s disease (Wu et al., 2016). Upregulated STIM2 senses ER Ca^2+^ content and leads to further dysregulation of SOCE (Wu et al., 2016). Efforts have been made to investigate the possibilities of targeting SOCE in Huntington’s disease. Wu and colleagues showed that the SOCE inhibitor, 6 amino 4 (4-phenoxyphenethylamino) (EVP4593), quinazoline normalized motor behavior in a fly model of Huntington’s disease. In addition, the application of EVP4593 provided protective effects in a glutamate toxicity assay in culture (Wu et al., 2016). Importantly, *in vivo* administration of EVP4593 in mice rescued age-dependent striatal spine loss (Wu et al., 2011). Ongoing efforts are directed to understanding the molecular targets of EVP4593. Interestingly, efforts have been made to elucidate SOCE machinery in Huntington’s disease using patient specific iPSC derived neurons. iPSCs were made from fibroblasts of Huntington’s disease patients and healthy donors and were subsequently differentiated into GABA-ergic neurons. SOCE was significantly enhanced in neurons derived from Huntington’s disease patients (Vigont et al., 2018). The same group of researchers later demonstrated that in a juvenile form of Huntington’s disease, there was increased SOCE that was mediated by STIM2 (Vigont et al., 2021). It is important to note that in this study all iPSCs were derived from a single donor of juvenile Huntington’s disease so additional studies are required.

Parkinson’s disease is currently the second most common neurodegenerative disease affecting more than 10 million people worldwide. Although currently the mechanisms for the loss of dopaminergic neurons in the substantia nigra remains unclear, it has been shown that mitochondrial dysfunction and alterations in Ca^2+^ homeostasis play a large part in the process. Two studies by Singh and colleagues demonstrated the functional role of SOCE in dopaminergic neurons in the substantia nigra. Interestingly, however, they reported that SOCE in these neurons were mediated by STIM1 and TRPC1 rather than Orai1 (Selvaraj et al., 2012; Sun et al., 2017). Dopaminergic neurons in the substantia nigra utilize Cav1.3 as the subunit for L type voltage gated calcium channel and following TRPC1 activation, L type Ca^2+^ current as well as the open probability of Cav1.3 were reduced (Sun et al., 2017). They also reported that silencing STIM1 and TRPC1 led to increased Cav1.3 current. Further, application of the neurotoxin that mimics Parkinson’s disease, 1-methyl-4-phenyl-1,2,3,6-tetrahyrdro-pyridine (MPTP), led to increased activity of Cav1.3, and decreased expression of TRPC1 and inhibited thapsigargin mediated STIM1-Cav1.3 interactions (Sun et al., 2017). Zhou *et al.* found that in fibroblasts from idiopathic Parkinson’s disease patients SOCE was impaired, but there was no change in STIM1 or Orai1 protein levels; however, the levels of PLA2g6 a Ca^2+^ independent phospholipase, which activates SOCE were significantly reduced (Zhou et al., 2016). Interestingly, mutations in PLA2g6 are associated with familial Parkinson’s disease and they found that knockout of PLA2g6 in mice resulted in Parkinson’s disease like symptoms. Loss of PLA2g6 was associated with dysfunctional autophagy in dopaminergic neurons, which was similar to that seen in Orai1-KO mice (Zhou et al., 2016). This study indicated that defects in PLA2g6 mediated SOCE, possibly via decreased Orai1 activity, could be a novel mechanism contributing to Parkinson’s disease.

Currently, most studies on STIM/Orai in Huntington’s and Parkinson’s disease have originated from only very few research groups and clearly much remains to be learned about the role of STIM and Orai proteins these diseases. For example, it remains unclear why STIM2 appears to play a predominant role in Huntington’s, whereas the STIM1-TRPC1 interactions have been identified in models of Parkinson’s disease. Moreover, it is surprising that there have been no reports of potential roles of Orai proteins in these diseases, even though STIM-Orai interactions are more commonly recognized as mediating SOCE. Future studies on iPSCs from Huntington’s and Parkinson’s disease patients represent a potentially valuable area of future investigation.

## 10 Role of STIM and Orai Proteins in Cellular Aging

While dysregulation in Ca^2+^ homeostasis is a hallmark of many age-related diseases such as cardiovascular and neurodegenerative diseases, the effect of aging itself on Ca^2+^ homeostasis has not received extensive investigation (Angenendt et al., 2020). Progerin, which is caused by a mutation in the Lamin A/C gene causes a premature aging syndrome called Hutchinson-Gilford progeria syndrome, and may also play a role in normal aging and in age related diseases (Ashapkin et al., 2019). Overexpression of progerin in myoblasts, resulted in the increased colocalization of STIM1 and Orai1 and enhanced SOCE (Wang et al., 2020), suggesting a potential link between aging and altered regulation of STIM and Orai mediated SOCE. STIM and Orai function have been widely studied in relation to the immune system and an age-related decline in immune system function is well established across many species (Nikolich-Zugich et al., 2012). Age-related decreases in Ca^2+^ signaling have been linked to dysfunction in aged lymphocytes. However, although the main Ca^2+^ signaling pathway in lymphocytes is mediated by STIM-activated Orai channels, little was known about the role during lymphocyte aging. Angenendt *et al.*, recently reported reduced SOCE in quiescent and activated lymphocytes from 18 to 24-month-old mice compared to 3–6-month-old mice (Angenendt et al., 2020). This reduction in SOCE was associated with reduced mRNA and protein levels of STIM1 and STIM2 under both conditions. On the other hand, in an *ex vivo* long term cell culture model designed to mimic aspects of aging, such as oxidative stress and DNA damage, the amplitude of SOCE was unchanged in aged lymphocytes although the Ca^2+^ dynamics following stimulation were altered (Rivet et al., 2016). The authors concluded that these changes in Ca^2+^ signaling were potentially a consequence of increased thiol oxidation of STIM1.

Aging is known to lead to vascular dysfunction in part by altered Ca^2+^ homeostasis and signaling in both VSMC as well as endothelial cells (Harraz and Jensen, 2021). However, even though STIM1 has been shown to be essential for regulating smooth muscle cell Ca^2+^ homeostasis and growth (Mancarella et al., 2013), little appears to be known about the role of STIM or Orai proteins in the aging process. Aortic medial degeneration, which is feature of both aortic dissection and aortic aneurysm is closely associated with aging (Nesi et al., 2009). Interestingly, microarray studies on human samples suggested that this might be due in part to lower STIM1 expression (Butt et al., 2016). In a murine model of aortic medial degeneration, inhibition of STIM1 exacerbated the development of medial degeneration (Hong et al., 2019). Of note, the effects of aging on VSMCs function appears to be dependent on specific vascular beds. For example, in 22-month-old rats, SOCE-induced vasoconstriction was enhanced in mesenteric arteries compared to 3-month-old rats; in contrast, it was decreased in the aorta. These changes in vasoconstriction were paralleled with changes in STIM1 and Orai1 protein expression in the different arterial beds (Yang et al., 2015). Despite the limited number of studies on the role of STIM1 on aging of the vasculature system, it appears complex and variable depending on the location of the vascular beds.

In skeletal muscle there have been some contradictory reports, where SOCE was maintained in muscles from aged mice despite reduced STIM1 levels (Edwards et al., 2011) while in another study SOCE was markedly reduced in aging muscle, but with no changes in either STIM1 or Orai1 expression (Zhao et al., 2008). It has also been reported that SOCE plays no role in the decrease in fiber force that occurs in senescent mouse muscle fibers (Payne et al., 2009). On the other hand, Thornton et al., concluded that impaired SOCE contributed to the decrease in contractile force characteristic of aging skeletal muscle (Thornton et al., 2011). Tubular aggregate myopathy (TAM) is a rare disorder skeletal muscle disorder associated with gain of function mutations in both STIM1 and Orai1. Tubular aggregates have also been described in extensor digitorum longus (EDL) muscles from 24-month-old male mice compared to 4–6 month-old mice (Boncompagni et al., 2020). The increase in tubular aggregates in aged muscle was associated with an increased accumulation of STIM1 and Orai1 and this was prevented by voluntary running from 9 to 24 months of age.

While there is some evidence that STIM1/Orai1 mediated SOCE is affected during normal aging the data are somewhat contradictory and gain or loss of function with aging appears to be tissue specific. It is also unknown whether aging leads to changes in the stoichiometry of STIM/Orai oligomers and if that would lead to changes in Ca^2+^ signaling. Given the relative paucity of studies examining how STIM and Orai proteins change during normal healthy aging it is premature to draw any firm conclusions. Nevertheless, the data that is available suggests that this could be a rich and important area for future research.

## 11 Conclusion

In the years since STIM1 and Orai1 were identified as the essential mediators of SOCE, the molecular mechanisms by which they facilitate Ca^2+^ entry into the cell have been explored in detail. While the canonical roles of STIM1 and Orai1 are broadly accepted in non-excitable cells, arguably there is much less consensus regarding the roles of their isoforms, STIM2, Orai2 and 3; moreover, the identification of an increasing number of splice variants further complicates the picture. In excitable cells, such as neurons and cardiomyocytes, the function of all STIM and Orai isoforms is much less clear; however, the use of cell-specific knockout or mutant models have clearly shown that they play an essential role in maintaining cellular homeostasis. The observations that STIM and Orai isoforms are subject to redox regulation has broad implications into how they might contribute to cellular dysfunction in the setting of oxidative stress.

Despite the rapid growth in our understanding of the role of STIM and Orai and the widely accepted notion that they are core elements of a highly evolutionarily conserved Ca^2+^ signaling pathway in all eukaryotes, numerous gaps in our knowledge remain. Such gaps include the understanding of the role of many post-translational modifications on the function (Johnson et al., 2022). In addition, considerable uncertainty remains regarding the composition of the Orai channel itself, its regulation by STIM isoforms and little is known how this stoichiometry changes in response to aging or pathology. The Orai channel has long been considered to be a hexamer composed of three Orai1 dimers with the ion pore located in the center and its activation occurring primarily as a result of STIM1 C-terminus coupling to the C-terminus of Orai1 (Derler et al., 2016a). While this has been a valuable working model it has contributed to our lack of understanding of the functions of other STIM and Orai isoform. The studies by Trebak and coworkers, which have suggested that all five STIM/Orai isoforms may work together to regulate the Ca^2+^ signaling responses (Yoast et al., 2020; Emrich et al., 2021) are intriguing and could help to explain the wide ranging differences in STIM-Orai Ca^2+^ signaling observed across different tissues and cell types. A better consensus as to the specific stoichiometry of STIM and Orai isoforms will be critical in helping to understand their role in aging and age-related diseases.

As we have described here, STIM and Orai proteins are increasing recognized as regulating cellular functions beyond the classic Ca^2+^ meditated transcription pathways. In addition to metabolic and mitochondrial regulation and cell survival, a protein complex including STIM1 is responsible for trafficking of early and late endosomes (Villari et al., 2020), consistent with its identification as a microtubule plus-end tracking protein. This could have wide ranging implications given the numerous fundamental functions of the endolysosomal system including metabolic signaling, plasma membrane remodeling autophagy and cell migration (Bonifacino and Neefjes, 2017). Clearly, given their potential role in cellular dysfunction in aging and age-related diseases, targeting STIM and Orai mediating signaling as a potential therapeutic target is of interest. However, as noted in the earlier section there are many knowledge gaps that currently limit the development of such therapeutics. However, these knowledge gaps provide considerable opportunity for future research and as they are filled will be improve our understanding of how defects in their function contribute to multiple disease processes, ultimately leading to development of novel therapeutic approaches to modulate their function.
